# Detecting the impact of subject characteristics on machine learning-based diagnostic applications

**DOI:** 10.1038/s41746-019-0178-x

**Published:** 2019-10-11

**Authors:** Elias Chaibub Neto, Abhishek Pratap, Thanneer M. Perumal, Meghasyam Tummalacherla, Phil Snyder, Brian M. Bot, Andrew D. Trister, Stephen H. Friend, Lara Mangravite, Larsson Omberg

**Affiliations:** 1 Sage Bionetworks, Seattle, USA; 2 Department of Biomedical Informatics and Medical Education, University of Washington, Seattle, USA; 3 4YouandMe, Seattle, USA; 4 Visiting Professor of Connected Medicine, Department of Psychiatry, Oxford University, Oxford, UK

**Keywords:** Preclinical research, Statistics

## Abstract

Collection of high-dimensional, longitudinal digital health data has the potential to support a wide-variety of research and clinical applications including diagnostics and longitudinal health tracking. Algorithms that process these data and inform digital diagnostics are typically developed using training and test sets generated from multiple repeated measures collected across a set of individuals. However, the inclusion of repeated measurements is not always appropriately taken into account in the analytical evaluations of predictive performance. The assignment of repeated measurements from each individual to both the training and the test sets (“record-wise” data split) is a common practice and can lead to massive underestimation of the prediction error due to the presence of “identity confounding.” In essence, these models learn to identify subjects, in addition to diagnostic signal. Here, we present a method that can be used to effectively calculate the amount of identity confounding learned by classifiers developed using a record-wise data split. By applying this method to several real datasets, we demonstrate that identity confounding is a serious issue in digital health studies and that record-wise data splits for machine learning- based applications need to be avoided.

## Introduction

The development of clinically actionable digital health assessments derived from high fidelity data obtained from wearables, smartphones, and in-home monitoring systems can transform the early diagnosis and treatment of health complications and diseases. Development of successful assessments requires strong interplay between experts from medical and analytical domains, as synthesizing high-dimensional sensor-based data often requires the use of sophisticated machine learning methods to derive robust inferences. Although few digital health measures have made it into clinical practice, early research have shown the clinical potential of digital biomarkers for assessment and remote management of diseases.^1–6^

As the sensors embedded in consumer grade devices get increasingly sophisticated at capturing data of high fidelity and frequency, machine learning models trained on such data are often able to uniquely identify the individual from whom each data stream is collected,^7^ illustrating the high sensitivity of sensor data to capture individualized “digital fingerprints” of the data contributors. In addition to privacy concerns, this also has major implications for development of diagnostic algorithms.

Development of diagnostic algorithms is typically performed by training a classifier in a “training dataset” and then estimating prediction performance in a second “test dataset”. Because classifiers will detect both biological and technical variation that are correlated with diagnostic state within the training dataset, any predictive approach needs to demonstrate model generalizability, e.g., high precision and recall, on a completely separate test dataset. Only classifiers that can effectively classify diagnoses across multiple datasets are deemed clinically useful. Because datasets are not always easy to obtain, initial development and evaluation of potential classifiers are often performed using data collected within a single study that is split into the training and test sets. In digital health applications, where it tends to be easy to collect dense longitudinal data from study participants, there are two main approaches for splitting the data into training and test sets:^8^ (i) “record-wise” split where each measurement or record is randomly split into training and test sets, allowing records from each subject to contribute to both the training and test sets; and (ii) “subject-wise” split where all the records of each subject are randomly assigned as a group to either the training set or to the test set.

Recently, Saeb et al.^8^ used a human activity recognition dataset to illustrate that the classification error rates estimated with record-wise and subject wise cross-validation could differ to a large extent. Furthermore, Saeb et al.^8^ demonstrated via simulation studies that (for diagnostic applications) splitting the data into training and test sets in a record-wise fashion can lead to massive underestimation of prediction error achieved by the machine learning algorithm. The problem arises because, in a record-wise split, the data from each subject can be in both the training and test sets so that the algorithm is not only learning about the outcome variable of interest but is also learning characteristics of the individual being measured. In a way, it is possible to build a digital fingerprint of individuals. In other words, the relationship between feature data and disease labels learned by the classifier is confounded by the identity of the subjects (from now on denoted as “identity confounding”). Since the easier task of subject identification replaces the harder task of disease recognition, classifiers trained on data split in a record-wise manner end up achieving overly optimistic prediction accuracy estimates,^8^ that are not disease relevant. Noteworthy, this practice is extremely common—a literature review by Saeb et al.^8^ found out that 28 out of 62 papers using repeated measurements for diagnostic purposes employed the record-wise data split. Furthermore, researchers in the field^9^ continue to advocate the use of record-wise data splits, arguing that the adoption of subject-wise data splits in heterogeneous datasets might lead to model under-fitting and larger classification errors. (See ref. ^9^ for a discussion, and different points of view, on this debate).

While the simulation approach adopted by Saeb et al.^8^ provides a clear illustration of the identity confounding issue, it only demonstrates the problem in synthetic data-sets and depends on a number of modeling choices (e.g., the relative strength of the disease and confounding signals, distributional assumptions for simulated data, etc) which might not be representable of actual data. Here, we propose a simple permutation approach that can be used on any dataset to quantify the amount of identity confounding learned by classifiers trained and evaluated using record-wise data splits.

Our non-parametric approach does not require any distributional assumptions, or a specific machine learning method and can be easily implemented on any dataset at hand. It can help to resolve some of the controversy around the adoption of record-wise versus subject-wise data splits^9^ and provides a valuable resource that editors and reviewers can use to stop over-optimistic estimates of predictive performance from being published. The ability to correctly assess the predictive performance of machine learning diagnostic systems has important practical implications since overestimated predictive accuracies can negatively impact decisions made by funding agencies, and might stimulate the deployment of untested health related apps, a current serious concern in the mobile health field.^10^

## Results

### Identity confounding in Parkinson’s disease digital health studies

We illustrate the application of our permutation approach using three real datasets from digital health studies of Parkinson’s disease, namely: the UCI Parkinson’s dataset;^11^ the UCI Parkinson’s Speech with Multiple Types of Sound Recordings dataset^12^ (from now on denoted as UCI Parkinson’s MSRD dataset); and the mPower dataset.^13^ For the mPower data, we focused on the voice and tapping activity tasks.

**Fig. 1:**
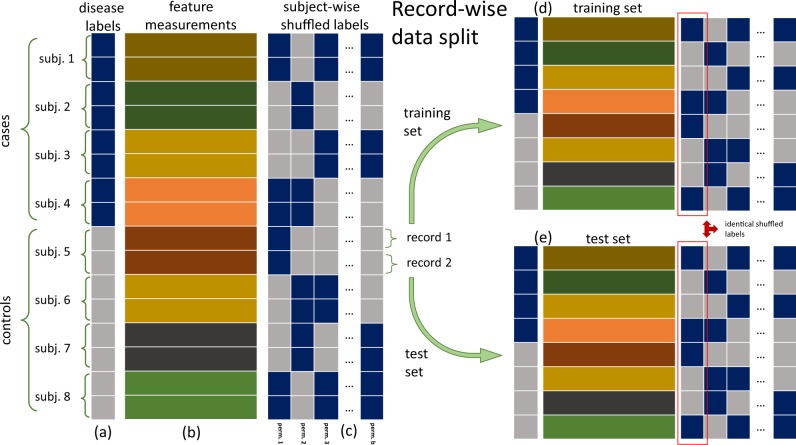
Permutation scheme to detect identity confounding. The schematic shows a toy example for a data-set with eight subjects (four cases and four controls), where each subject contributed two records. **a** and **b** show, respectively, the disease label vector and the feature matrix. **c** Shows distinct “subject-wise random permutations” of the disease labels, where the permutations are performed at the subject level, rather than at the record level (so that all records of a given subject are assigned either “case” or “control” labels). For example, in the first permutation, the labels of subjects 2 and 3 changed from “case” to “control”, the labels of subjects 5 and 8 changed from “control” to “case”, and the labels of subjects 1, 4, 6, and 7 remained the same. (Note that for each subject, the labels are changed across all records). The subject-wise label permutations destroy the association between the disease labels and the features, making it impossible for a classifier trained with shuffled labels to learn the disease signal. Adopting the record-wise data split strategy, with half of the records assigned to the training set (**d**), and the other half to the test set (**e**), we have that both training and test sets contain 1 record from each subject. Most importantly, in each permutation the shuffled labels of each subject are the same in both the training and test sets. For instance, in the first permutation (highlighted by the red boxes in **d**, **e**) we have that the shuffled labels of subjects 1 to 8, namely, “case”, “control”, “control”, “case”, “case”, “control”, “control”, “case”, are exactly the same in the training and test sets. Consequently, any classifier trained with the shuffled labels will still be able to learn to identify individuals, even though it cannot learn the disease signal

The basic idea behind our permutation approach is to generate a null distribution (for a chosen predictive performance metric) where the association between the disease labels and the features is destroyed while the associations between the features and the subject identities is still preserved. (See “Methods” for details and Fig. 1 for an illustration of the permutation scheme.) In practice, the presence of identity confounding can be informally inferred from the location of the permutation null distribution. More specifically, the ability to identify subjects allows the classifier to make correct predictions at a higher rate than a random guess, so that the permutation null distribution is located away from the baseline random guess value. (For instance, for the area under the receiver operating characteristic curve—AUC—metric, the baseline random guess value corresponds to a 0.5 AUC score. Therefore, the presence of identity confounding can be detected whenever the permutation null distribution is located at an AUC value larger than 0.5).

**Fig. 2:**
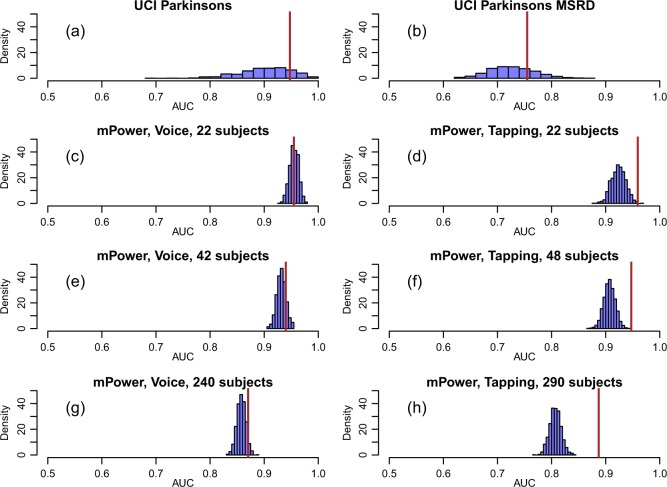
Identity confounding in digital health data sets. In all panels, the permutation null distribution is represented by the blue histogram while the observed AUC value is represented by the brown line. In all panels the permutation null distribution is shifted away from 0.5—the baseline random guess value for the AUC metric. **a**, **b** Show the results for the UCI Parkinson’s and UCI Parkinson’s MSRD data sets, respectively. Note the larger spread of the permutation null distributions (compared to the remaining panels). Panel **c** shows the results for the mPower voice data based on 22 subjects. Note that the observed AUC falls right in the middle of the permutation null distribution. Panel **d** shows the results for the tapping task based on 22 subjects. In this case, however, the observed value falls in the tail of the null distribution. **c**, **e**, and **g** compare the results for the mPower voice data across increasing numbers of subjects (namely, 22, 42, and 240 subjects). **d**, **f**, **h** show the analogous comparison for the mPower tapping data (based on 22, 48, and 290 subjects, respectively). The results were generated using the random forest classifier, and were based on 1000 permutations. See the Methods section for a description of the permutation scheme used to generated the permutation null distributions

The analysis of the three digital health datasets described above uncovered strong amounts of identity confounding. Figure 2 reports the results based on the AUC metric, and shows that the permutation null distributions were centered far away from 0.5 for the UCI Parkinson’s dataset (panel a), the UCI Parkinson’s MSRD dataset (panel b), and the mPower dataset (panels c to h). The identity confounding issue was particularly strong for the classifier built from the voice data collected from a subset of 22 participants from the mPower study (Fig. 2c) where the permutation null distribution (which captures the identity confounding) was centered at a 0.95 AUC score.

In addition to detecting identity confounding, the permutation null distributions in Fig. 2 can also be used to check if the classifier is learning the disease signal in addition to learning subject specific signals. This can be done by simply comparing the location of the permutation null distributions relative to the observed AUC score computed using the original (unshuffled) data. (Note that the proportion of permutations showing a value equal or greater than the observed AUC corresponds to a permutation p-value to test if the classifier is learning the disease signal in the presence of identity confounding). For instance, for the classifier built with the voice data based on a small subset of subjects (Fig. 2c), the fact that the observed AUC falls right in the middle of the permutation null indicates that the classifier is mostly performing subject identification with no or little additional learning of disease. On the other hand, the fact that the observed AUC falls at the tail of the permutation null distribution for the classifier built with the tapping data (Fig. 2d) indicates that it was still able to learn the disease signal in addition to the subject identity signal. (As described in the Supplementary Methods, the number of permutations showing AUC scores above the observed AUC corresponds to a permutation p-value to test if the classifier has learned the disease signal in addition to the identity signal).

In order to evaluate the influence of the number of subjects on the classifier’s ability to learn the identity confounding, we considered three subsets of participants composed of increasing numbers of subjects in the mPower data. As expected, the permutation null distributions tended to be located at smaller AUC values, as the number of subjects used to train the classifiers increased (since a larger number of subjects makes it more difficult for the classifier to identify individual subjects). Figure 2c, e, g shows the comparison for the mPower voice data, while Fig. 2d, f, h shows the analogous comparison for the mPower tapping data. Overall the trend is the same for both voice and tapping but with tapping capturing the subject identity signal to a slightly lesser degree (the permutation null distributions tend to be located at lower AUC values for the tapping data than for the voice data), and are better able to perform disease recognition (the AUC values are located farther away in the tails of the permutation null distributions).

Finally, note that the larger spread of the permutation null distributions for the UCI datasets (Fig. 2a, b) is due to the smaller test set sizes available for these datasets.

## Discussion

In this paper, we employ a permutation approach to quantify identity confounding in clinical machine learning applications where repeated measures or records have been captured on each subject. We illustrate the application of the proposed tests with real data from Parkinson’s disease digital studies.^11–14^ In all these examples, the goal is to predict if a given subject has Parkinson’s or is a “control” subject. Observe that, in these applications, each subject has a single label type (e.g., each subject is either a Parkinson’s or a control subject). There are, nonetheless, other classification problems where each subject can have multiple labels. For instance, diagnostic applications aiming to classify disease severity (e.g., mild versus severe disease state) might actually contain data from the same subject collected at different times under different disease severity states. (Activity recognition is another example, where the goal is to classify distinct behaviors performed by each subject at different times, such as walking, running, sitting down, etc.). While the permutation approach described in this paper is not directly applicable to these “multiple labels per subject” problems, restricted permutations^15^ might represent an alternative solution in this setting (this extension is nonetheless left as future work). Observe, as well, that while our illustrations are all based on the AUC metric, our permutation approach can be implemented with any other performance metrics. Furthermore, the approach can be directly extended to multi-class classification tasks (with a single label per subject), and can also be adapted for regression tasks.

While in this paper we present the permutation approach in an informal (although precise) style, it can be used to derive formal hypothesis tests to evaluate if a classifier has learned the disease signal in the presence (or absence) of identity confounding. Furthermore, a simple extension of the permutation approach can also be used to perform a statistical test to detect identity confounding per se. For the sake of the more technically inclined reader, the Supplementary Methods presents: (i) a more technical description of our methods; (ii) several illustrative examples using synthetic data; (iii) a more detailed analysis of the mPower data; and (iv) a simulation study showing the statistical validity of our permutation tests.

Our analyses of the voice and tapping data, collected by the mPower study, showed undeniable evidence of a high degree of identity confounding for classifiers built using record-wise data splits. Although it is true that our results also showed evidence of model under-fitting for classifiers trained with subject-wise data splits (see the Supplementary Methods for the subject-wise analyses), we point out that model under-fitting can be ameliorated by simply increasing the number of subjects used in the analyses. (The rationale is that, as the number of subjects used to train the classifier increases, the chance that the training set is missing a critical part of the pattern that relates features to disease labels decreases, so that the classifier has a better chance to generalize to new unseen cases, even when the data is fairly heterogeneous). But, most importantly, our results do not support the hypothesis that model under-fitting (alone), rather than identity confounding, could explain the discrepancy in classification performance between the record-wise and subject-wise strategies.^9^

Furthermore, in the Supplementary Methods, we illustrate with simulations that many different sources of heterogeneity in the feature data can easily give rise to identity confounding. These results clearly show that identity confounding is a serious issue in digital health studies and that record-wise data splits should be avoided. In agreement with Saeb et al.,^8^ and others^12,16,17^ our recommendation is that subject-wise data splits (where all records of a given participant are assigned either to the training or to the test set, but not to both) should be used in machine learning diagnostic applications. Clearly, any source of identity confounding is automatically neutralized by the subject-wise data splitting strategy.

The use of record-wise data splits can lead to the overestimation of the real accuracy of machine learning-based diagnostic systems. This practice might lead to disinformation of the scientific community, funding of follow-up research studies based on false promises of good performance, or even to increased misdiagnosis of patients if systems evaluated with record-wise data splits get deployed in clinical practice. Scientists in the field continue to advocate for the use of record-wise splitting, particularly for applications with small sample sizes such as is seen with emerging data modalities or those that are difficult to collect. This tool is designed to help those scientists—and the larger field—evaluate diagnostic classifiers developed in that manner. We believe that the availability of a simple, yet, rigorous method able to demonstrate the presence and strength of identity confounding will help researchers to evaluate the strength of this common artifact in digital health diagnostic applications. In particular, reviewers of manuscripts and research grants now have a new tool to objectively evaluate studies that employ record-wise data splits.

Finally, we point out that detection of identify confounding is one of several important assessments that must be performed when evaluating the reliability of a classifier. In a previous contribution,^18,19^ we proposed a distinct permutation approach to quantify the contribution of demographic confounders such as age, gender, etc, to the predictive performance of a classifier.

## Methods

### Datasets

#### UCI Parkinson’s dataset

This dataset (https://archive.ics.uci.edu/ml/datasets/parkinsons) is composed of 22 features extracted from voice measurements^11,20^ of 32 subjects (24 PD cases and 8 healthy controls). Each subject contributed 6 or 7 records, generating a total of 195 records.

#### UCI Parkinson speech with multiple types of sound recordings dataset

This dataset (https://archive.ics.uci.edu/ml/datasets/Parkinson+Speech+Dataset+with++Multiple+Types+of+Sound+Recordings) is composed of 26 linear and time frequency based features, extracted from 26 voice measurements^12^ including sustained vowels, numbers, words and short sentences. The training dataset is composed of 1040 records, contributed by 20 PD cases and 20 healthy controls (each subject contributed 26 records). Note that while an additional test set composed of a separate cohort of 28 PD cases is available in this dataset, we restricted our analyses to the training data.

#### mPower dataset

This data set contains voice, tapping, walk, and rest activity tasks collected by smartphones, contributed by thousands of self-reported PD case and control participants.^13^ For our illustrations we focus on the voice and tapping data, and evaluate three subsets of participants for each task. For the voice task we evaluated: (i) a subset of 11 PD cases and 11 controls that contributed at least 100 records; (ii) a subset of 21 PD cases and 21 controls that contributed at least 50 records; and (iii) a subset of 120 PD cases and 120 controls that contributed at least 10 records. For the tapping task we evaluated: (i) a subset of 11 PD cases and 11 controls that contributed at least 100 records; (ii) a subset of 25 PD cases and 24 controls that contributed at least 50 records; and (iii) a subset of 145 PD cases and 145 controls that contributed at least 10 records. All case and control participants were males, and were first matched by age (using exact matching), and then by education level (using nearest neighbor matching^21^) whenever there were multiple cases with the same age of a control, or vice-versa. (In the event that ties persisted after the application of this second matching criterion, we randomly selected one participant to perform the matching.) The analyses were based on 13 voice features and 41 tapping features proposed in the literature.^22,23^ Participants of the mPower study completed an interactive, in-app informed consent process that included a quiz on the risks, benefits, and options for study participation and sharing. The study was approved by Western Institutional Review Board (WIRB protocol #20141369), and registered at ClinicalTrials.gov (identifier #NCT02696603).

### Model training and evaluation

In all illustrations, we adopted the area under the receiver operating characteristic curve (AUC) as the performance metric, and employed the random forest classifier^24^ implemented in the randomForest R package,^25^ using the default tuning parameter specifications. In all analyses, each dataset was randomly split into a training and test set of roughly equal sizes. (The Supplementary Methods also presents the analyses of the mPower data based in data splits assigning 90% of the records to the training set, and 10% to the test set (Supplementary Figs 14 and 15). As described in more detail in the Supplementary Methods, increasing the amount of training data tends to improve the ability of the classifiers to identify the subjects).

The AUC calculations were performed according to the following steps. First, we train the classifier using the training set. Second, we use the test set features to generate the class membership probabilities for the test set label predictions. Third, we use the positive class probabilities together with the test set labels to compute the AUC score using the pROC^26^ R package. Note that the observed AUC score is computed as described above using the original (i.e., unshuffled) data, while the permutation null distribution for the AUC metric is computed applying these three steps to training and test sets with shuffled label data (as described in the next section).

### Generation of the permutation null distribution

The basic idea is to generate a permutation null distribution for a chosen predictive performance metric (e.g., AUC), where the association between the disease labels and the features is destroyed while the associations between the features and the subject identities is still preserved. To this end, we generate a permutation null distribution as follows. First, we shuffle the disease labels of each subject as a block across all records, so that all records of a given subject are assigned either “case” or “control” labels during the permutation process. By shuffling the disease labels in this subject-wise fashion we destroy the association between the disease labels and the features, hence removing the ability of the classifier to learn disease signal. Second, we split the data in a record-wise manner so that part of the records of each subject are assigned to the training set, while the remaining records are assigned to the test set. Note that the shuffled labels of each subject are the same in both the training and test sets. As a consequence, any classifier trained with the shuffled labels will still be able to learn to identify individuals, even though it cannot learn the disease signal. Figure 1 provides a toy illustrative example.

Algorithm 1 in the Supplementary Methods describes in detail the generation of the Monte Carlo permutation null distribution (blue histograms in Fig. 2). Here, we present a summary of the process. Basically, the permutation null is computed as follows: (i) generate a random split of the data by generating two sets of indexes (training and test indexes) representing which data points will be assigned to the training and test sets, respectively; (ii) split the feature data into training and test sets using the data-split indexes generated in step i; (iii) generate a subject-wise shuffled version of the label data; (iv) split the shuffled label data into training and test sets using the data-split indexes generated in step i; (v) train a classifier using the training features and training shuffled labels; (vi) evaluate the performance of the classifier on the test set (where the test set features are used to generate predictions that are compared to the test set shuffled labels); (vii) record the value of the performance metric calculated on step vi; and (viii) repeat steps (iii) to (vii) a large number of times. The procedure used to generate the subject-wise label permutations in step (iii) is described in detail in Algorithm 2 in the Supplementary Methods.

## Supplementary information

Supplementary materials

## Data Availability

The UCI Parkinson’s dataset is available from the UCI repository^27^ at: https://archive.ics.uci.edu/ml/datasets/parkinsons. The UCI Parkinson’s MSRD dataset is available from the UCI repository^27^ at: https://archive.ics.uci.edu/ml/datasets/Parkinson+Speech+Dataset+with++Multiple+Types+of+Sound+Recordings. The mPower dataset supporting the results presented on this article is available from Synapse at: 10.7303/syn11623804. To gain access to the mPower dataset, researchers must follow the instructions described in the wiki, https://www.synapse.org/#!Synapse:syn4993293/wiki/247860. This qualification process will provide access to the entire mPower data,^13^ in addition to the subset used in this article.

## Code availability

The R code used in this paper is available in github: https://github.com/Sage-Bionetworks/mHealthIdentityConfounding
