# Supplementary material for: Detecting the impact of subject characteristics on machine learning-based diagnostic applications
Source: NPJ Digit Med. 2019 Oct 11;2:99. doi: 10.1038/s41746-019-0178-x (PMC6789029; doi:10.1038/s41746-019-0178-x)
Supplement: Supplementary file 1 — Supplementary materials [file 41746_2019_178_MOESM1_ESM.pdf]

Supplementary materials for "Detecting the impact of subject characteristics on machine learning-based diagnostic applications"

by Elias Chaibub Neto*,[1], Abhishek Pratap[1,2], Thanneer M Perumal[1], Meghasyam Tummalacherla[1], Phil Snyder[1], Brian M Bot[1], Andrew D Trister[1], Stephen H Friend[1], Lara Mangravite[1], and Larsson Omberg[1]

[1] Sage Bionetworks, Seattle
[2] Department of Biomedical Informatics and Medical Education, University of Washington, Seattle
* elias.chaibub.neto@sagebase.org

1 Motivation

Saeb *et al*[1] used simulations to show that, in diagnostic machine learning applications, having data of each subject randomly assigned to both training and test sets (record-wise data split) can lead to massive underestimation of the cross-validation prediction error, due to the presence of subject "identity confounding" caused by the classifier's ability to identify subjects, instead of recognizing disease. To solve this problem, the authors recommended the random assignment of the data of each subject to either the training or the test set (subject-wise data split). The adoption of subject-wise split has been criticized by the first author of Little *et al*[2], on the basis that it can violate assumptions required by cross-validation to consistently estimate generalization error. In particular, adopting subject-wise splitting in heterogeneous data-sets might lead to model under-fitting and larger classification errors. Hence, the first author of Little *et al*[2] argues that perhaps the over-estimation of prediction errors with subject-wise cross-validation, rather than underestimation with record-wise cross-validation, is the reason for the discrepancies between prediction error estimates generated by the two splitting strategies. Here, we describe statistical tests that can be used to evaluate the merits of record-wise and subject-wise data splits under general statistical dependencies and distributional structures of the data, and can be used to answer this question.

Based on the permutation scheme described in the main text, here we describe two formal permutation tests. (The first test allows us to determine whether the classifier is performing disease recognition, even when identity confounding is present. The second test, builds on the first, and allows us to test for the presence of identity confounding per se.) We illustrate the application of the proposed permutation tests with synthetic data examples, and provide a more detailed analysis of the mPower study data[3, 4]. The synthetic data examples illustrate that identity confounding can arise for many different reasons, including serial dependency in the feature data of each participant (i.e., serially associated records), as well as, due to location and scale differences in the feature distributions across the subjects (even when the longitudinal feature measurements are independent of each other). The real data examples illustrate that a high degree of identity confounding can be present in digital health applications.

2 The proposed permutation tests

Before describing the permutation tests, we first introduce some notation. Throughout the text we let $s = \{1, 2, \ldots, S\}$ index the subjects, $r_s = \{1, 2, \ldots, R_s\}$ index the records across subject s, and $f = \{1, 2, \ldots, f\}$ index the features. $\boldsymbol{X}$ represents the $\sum_s R_s \times f$ matrix of feature data, with columns indexing the features, and rows indexing the records across all S subjects, while $\boldsymbol{y}$ represents the corresponding vector of disease labels, with dimension $\sum_s R_s \times 1$. We let m represent an arbitrary classification performance metric, and reserve p to represent the number of permutations employed

in our tests. We let A and B represent the events,

$$A \equiv \text{the classifier is performing disease recognition,} \tag{1}$$

$$B \equiv \text{the classifier is performing subject identification,} \tag{2}$$

and adopt standard set theory notation where: E^c represents the complement of event E; $\emptyset$ represents the empty set; Ω represents the universe; and the symbols $\cup$ and $\cap$ represent the union and intersection of events, respectively.

2.1 Assessing whether the classifier is performing disease recognition

In order to evaluate whether the classifier is performing disease recognition (even when identity confounding is present) we need to generate a permutation null distribution where the association between the disease labels and the features is destroyed while the association between the features and the subject identities is still preserved. To this end, we generate a permutation distribution by shuffling the disease labels in a subject-wise fashion, as described in Algorithms 1 and 2, and test the hypotheses,

$$H_0^* : \text{the classifier is not performing disease recognition,}$$

$$H_1^* : \text{the classifier is performing disease recognition}$$

Algorithm 1 Permutation null distribution for performance metric m

Input: Number of permutations, p; feature data matrix, $\boldsymbol{X}$; label data vector, $\boldsymbol{y}$; training and test set indexes, i_{train}, i_{test}; subject label vector, $\boldsymbol{z}$
Split $\boldsymbol{X}$ into training and test feature sets, $\boldsymbol{X}_{train} \leftarrow \boldsymbol{X}[i_{train},]$, $\boldsymbol{X}_{test} \leftarrow \boldsymbol{X}[i_{test},]$
for $i = 1, 2, \ldots, p$ **do**
    Generate a subject-wise shuffled version of the label data, $\boldsymbol{y}^* \leftarrow \text{SubjectWiseShuffle}(\boldsymbol{y}, \boldsymbol{z})$ (see Algorithm 2 for details)
    Split $\boldsymbol{y}^*$ into training and test label sets, $\boldsymbol{y}^*_{train} \leftarrow \boldsymbol{y}^*[i_{train}]$, $\boldsymbol{y}^*_{test} \leftarrow \boldsymbol{y}^*[i_{test}]$
    Train a classifier on the $\boldsymbol{X}_{train}$ and $\boldsymbol{y}^*_{train}$ data
    Evaluate the classifier performance on the $\boldsymbol{X}_{test}$ and $\boldsymbol{y}^*_{test}$ data
    Record the value of the performance metric, m_i^*, on the shuffled data
end for
Output: m_1^*, m_2^*, ..., m_p^*

Algorithm 2 Subject-wise label permutation (`SubjectWiseShuffle`)

Input: Label vector, $\boldsymbol{y}$; subject label vector, $\boldsymbol{z}$, (i.e., a vector of length equal to the number of subjects where each entry corresponds to 1 if the subject has the disease, or to 0 if the subject is a control)
Generate a permutation, $\boldsymbol{z}^*$, of the subject label vector
Initialize the permuted label vector, $\boldsymbol{y}^* \leftarrow \boldsymbol{y}$
for $s = 1, 2, \ldots, S$ **do**
    Get the index set i_s (namely, the positions, in vector $\boldsymbol{y}$, of all records provided by subject s)
    Replace the disease label by the permuted label across all records contributed by subject s in the permuted label vector, $\boldsymbol{y}^*[i_s] \leftarrow \boldsymbol{z}^*[s]$
end for
Output: $\boldsymbol{y}^*$

Note that because we are using the performance metric, m, as the test statistic of the permutation test (and m is able to capture the contributions of both disease recognition and subject identification to the classification performance), we have that the alternative hypothesis H_1^* is equivalent to "the classifier is performing disease recognition irrespective of whether it is also doing subject identification or not".

A permutation p-value for testing H_0^* is computed as the proportion of times that the performance metric computed in shuffled label data was better than the performance metric computed with the original (un-shuffled) labels, m_o. For instance, for a performance metric such as the area under the receiver operating characteristic curve (AUC), where larger values indicate better classification performance, the permutation p-value can be estimated as

$$\sum_{i=1}^{p} \mathbb{1}\{m_i^* \geq m_o\}/p \ , \tag{3}$$

where $\mathbb{1}$ represents the indicator function, assuming value 1, when $m_i^* \geq m_o$, and 0, otherwise. (For performance metrics where smaller values indicate better classification performance, such as misclassification rate, the permutation p-value is given by $\sum_{i=1}^{p} \mathbb{1}\{m_i^* \leq m_o\}/p$.)

Note that when the data is split in a record-wise fashion, and identity confounding is present, the disease recognition null distribution will be centered away from the baseline random guess value (e.g., 0.5 when adopting the AUC) since in the generation of the null distribution the label data is split into training and test sets only after the labels are shuffled (see steps 4 to 7 of Algorithm 1) so that both training and test sets have the same shuffled labels (as illustrated in Figure 1 in the main text). As a consequence, the presence of identity confounding will show up as an ability to classify the permuted labels with an accuracy that is better than a random guess (since even though the shuffling of labels prevent the algorithm from performing disease recognition, it is still able to perform subject identification). For example, when adopting the AUC metric, and in the presence of strong identity confounding, the disease recognition null will be centered at AUC values closer to 1 than to 0.5. Hence, the disease recognition null distribution not only allows us to test whether the classifier is performing disease recognition, but also informally allows us to infer the presence of identity confounding by simply inspecting how far it is centered from the baseline random guess value. Finally, note that the proposed disease recognition permutation test assumes that the disease labels are exchangeable across subjects (in a subject-wise fashion) under the null H_0^*.

2.2 Detecting identity confounding

As described above, the presence of identity confounding will shift the disease recognition null distribution away from the baseline random guess value. Hence, the median of the disease recognition null distribution,

$$\tilde{m}^* = \text{median}(m_1^*, m_2^*, \ldots, m_p^*) \ , \tag{4}$$

represents a natural statistic to quantify identity confounding alone, as it measures the contribution of identity confounding to the classifier's predictive ability, after the algorithm's ability to recognize disease has been neutralized by the subject-wise shuffling of disease labels. (Note that while other statistics such as the mean of the disease recognition null distribution could be used to quantify identity confounding, we prefer the more robust median metric since the distribution can be asymmetric.)

In order to test whether the classifier is performing subject identification, we need to generate a permutation null distribution (for the $\tilde{m}^*$ statistic) where the association between the subject identities and the features is broken. To this end, we shuffle the feature data in a record-wise fashion, before computing the $\tilde{m}^*$ statistic, as described in Algorithm 3.

Note that because the record-wise shuffling of the feature data also breaks the association between the features and the disease labels, the identity confounding null distribution will always be centered around the baseline random guess value (0.5 for the AUC metric).

Algorithm 3 Permutation null for detecting identity confounding

```
1: Input: Number of feature permutations, p; number of label permutations, p_l; feature data matrix, X; label data vector, y; training and test set indexes, i_train, i_test
2: for i = 1, 2, ..., p do
3:     Generate a record-wise shuffled version of the feature data, X*
4:     Split X* into training and test sets, X*_train ← X*[i_train, ], X*_test ← X*[i_test, ]
5:     for j = 1, 2, ..., p_l do
6:         Generate a subject-wise shuffled version of the label data, y* (see Algorithm 2 for details)
7:         Split y* into training and test label sets, y*_train ← y*[i_train], y*_test ← y*[i_test]
8:         Train a classifier on the X*_train and y*_train data
9:         Evaluate the classifier performance on the X*_test and y*_test data
10:        Compute the value of the performance metric, m**_j, on the shuffled data
11:    end for
12:    Record the median of the perf. metric distr., m̃**_i = median(m**_1, m**_2, ..., m**_{p_l})
13: end for
14: Output: m̃**_1, m̃**_2, ..., m̃**_p
```

For performance metrics where larger values indicate better classification performance, the permutation p-value for testing the hypotheses,

$$H_0^{**} : \text{the classifier is not performing subject identification}$$
$$H_1^{**} : \text{the classifier is performing subject indentification,}$$

is estimated as,

$$\sum_{i=1}^{p} \mathbb{1}\{\tilde{m}_i^{**} \geq \tilde{m}^*\}/p \ , \qquad (5)$$

where $\tilde{m}^*$ represents the observed value of the test statistic, computed on the original (un-shuffled) feature data, and defined in equation (4), whereas $\tilde{m}_i^{**}$ represents the statistic computed on the shuffled feature data, as described in step 12 of Algorithm 3. (Otherwise, it is computed as $\sum_{i=1}^{p} \mathbb{1}\{\tilde{m}_i^{**} \leq \tilde{m}^*\}/p$.)

Note that when we shuffle the feature data in a record-wise fashion we, of course, break the connection between the feature data and both the subject identities and the disease labels. We point out, nonetheless, that the null hypothesis H_0^{**} is given by $H_0^{**} : B^c$ and not by $H_0^{**} : B^c \cap A^c$ because, by construction, our test statistic only captures the contribution of the subject identification to the classifier's performance (where events A and B are defined in eqs 1 and 2). Observe, as well, that the identity confounding permutation test assumes the exchangeability of the feature data (in a record-wise fashion) under the null H_0^{**}.

One important drawback of this permutation test is the high amount of computation required to generate the null distribution. We point out, however, that for the particular case where the AUC metric is adopted to evaluate the classifier performance, it is sometimes possible to infer the presence of identity confounding using an analytical approach without having to run the permutation test.

2.3 An analytical shortcut for detecting identity confounding, based on the AUC metric

Here, we describe an alternative approach to detect identity confounding. While it is not a proper statistical test, it provides a statistic similar to a p-value (denoted a "pseudo p-value") which can still be useful in practice. But, before presenting the pseudo p-value statistic, we first describe an additional statistical test that helps understand the pseudo p-value approach.

Consider a third permutation test, where we adopt the AUC as the test statistic, and generate a null distribution by shuffling the disease labels in a record-wise fashion. In this case, because the AUC metric is able to capture the contributions of both disease recognition and subject identification to the classification performance, and the record-wise shuffling of labels breaks the connection between the disease labels and both the feature data and the subject identities, we have that the permutation procedure is now testing the null hypothesis $A^c \cap B^c$ against the alternative $A \cup B$ (where events A and B are defined in eqs 1 and 2). Hence, the permutation p-value for testing this third set of hypothesis,

$$H_0^{***} : \text{the classifier is not performing disease recognition and subject identification,}$$
$$H_1^{***} : \text{the classifier is performing disease recognition or subject identification,}$$

is computed as,

$$\sum_{i=1}^{p} \mathbb{1}\{\text{auc}_i^* \geq \text{auc}_0\}/p \ , \tag{6}$$

where auc_0 represents the AUC value computed from the original data.

For this statistical test, nonetheless, an analytical solution is available. Explicitly, it has been shown[5] that, when there are no ties in the predicted class probabilities used for the computation of the AUC, the test statistic of the Mann-Whitney U test (U) is related to the AUC statistic by, $U = n_n\, n_p(1 - \text{AUC})$, where n_n and n_p represent the number of negative and positive labels in the test set (see section 2 of reference[6] for details).

In the presence of ties, the p-value can be computed as the left tail of the asymptotic approximate null,

$$U \approx \text{N}\left(\frac{n_n\, n_p}{2}\ , \ \frac{n_n\, n_p(n+1)}{12} - \frac{n_n\, n_p}{12\, n\,(n-1)} \sum_{j=1}^{\tau} t_j(t_j - 1)(t_j + 1)\right) \ , \tag{7}$$

where $n = n_n + n_p$, τ is the number of groups of ties, and t_j is the number of ties in group j[6].

Alternatively, we can get the p-value as the right tail probability of the corresponding AUC null, $\text{AUC} \approx \text{N}(0.5\, , \, \phi^2)$,

$$\text{p-value} = 1 - \Phi\left(\frac{\text{auc}_0 - 0.5}{\phi}\right) \ , \tag{8}$$

where $\Phi()$ represents the cumulative distribution function of a standard normal distribution, and,

$$\phi^2 = \frac{n+1}{12\, n_n\, n_p} - \frac{1}{12\, n_n\, n_p\, n\,(n-1)} \sum_{j=1}^{\tau} t_j(t_j - 1)(t_j + 1) \ . \tag{9}$$

Here, we propose to use the pseudo p-value statistic,

$$\text{pseudo p-value} \equiv 1 - \Phi\left(\frac{\tilde{\text{auc}}_0^* - 0.5}{\phi}\right) \ , \tag{10}$$

as an alternative measure for identity confounding, where $\tilde{\text{auc}}_0^*$ corresponds to the median of the disease recognition null distribution computed using the AUC metric.

Note that this procedure does not correspond to a proper statistical test, since it compares the value of one test statistic against the null distribution of a different test statistic, effectively performing an "apples-to-bananas" comparison (namely, it compares the median of the AUC metric under the disease recognition null distribution against the distribution of the AUC metric under H_0^{***}). (The correct comparison is the one described in the previous section, where we compare the observed median statistic $\tilde{\text{auc}}_0^*$ to the null distribution for the same statistic, generated according to Algorithm 3.)

In any case, the pseudo p-value might be still useful in practice, because the null distribution of the AUC statistic under H_0^{***} will always have a larger spread than the null distribution of the median AUC statistic under H_0^{**}[1]. Hence, if the pseudo p-value is already very small, there is no need to perform the computationally expensive permutation test for detecting identity confounding. We point out, however, that a non-significant pseudo p-value does not necessarily mean lack of identity confounding, and that, in this case, it is necessary to compute the permutation p-value since identity confounding can still be present when the pseudo p-value suggests it is not.

3 Illustrative examples

We illustrate the application of our permutation tests using synthetic and real data-sets, split into training and test sets according to both the record-wise and subject-wise data split strategies. (Note that we apply the identity confounding permutation test to both record-wise and subject-wise data splits for illustrative purposes. In practice, however, it is not necessary to test for identity confounding when adopting subject-wise data splits, as the influence of individual characteristics are automatically neutralized by the subject-wise strategy.) In all illustrations, we adopt the AUC as the performance metric, and employ the random forest classifier[7] implemented in the `randomForest` R package[8], using the default tuning parameter specifications.

3.1 Synthetic data-sets

In this section, we illustrate the presence of identity confounding using synthetic data examples. Our goal is to show that many different sources of heterogeneity in the feature data can easily give rise to identity confounding (and that our permutation tests are able to detect it). We do not make any claims that the particular model adopted to simulate the feature data is able to closely reproduce the dependency/distributional structures observed in real mobile data (as any simulated example will always be artificial to some extent).

When the training and test sets are obtained by record-wise data split, identity confounding can arise due to statistical dependencies in the feature data of each participant (e.g, serial association in longitudinal data), as well as, due to differences in the average or variance of the feature distributions

[1]The following rational shows why the spread of the permutation null distribution generated under H_0^{**} is smaller than the spread of the permutation null generated under H_0^{***}. Note that in order to generate a permutation distribution under the null hypothesis H_0^{**} (i.e., the classifier is not performing subject identification) we need to employ Algorithm 3, which performs a "double-shuffling" of the data (implemented with an internal for-loop nested inside an external for-loop). Observe that for each of the p iterations of the external for-loop (step 2 of Algorithm 3) the feature data is first shuffled in a record-wise manner (step 3). Observe, as well, that for each shuffled version of the feature data, the internal for-loop (step 5) performs p_l subject-wise shuffles of the label data, and for each of these p_l iterations the AUC is computed using the record-wise shuffled features and subject-wise shuffled labels (step 10). Finally, the median of the p_l AUC scores generated in the internal for-loop is computed at each of the p iterations of the external for-loop (step 12 od Algorithm 3). Now, note that because the record-wise shuffle of the feature data already breaks the association between the disease labels and the subject identity it turns out that the distribution of the AUC scores generated by the internal for-loop (steps 5 to 11) will be centered around 0.5. Hence, the median of these p_l AUC scores (step 12) will always be very close to 0.5. Therefore, it follows that the distribution of the median AUC scores (generated from the p iterations of the external for-loop) will always show a smaller spread around 0.5 than each of the AUC distributions generated using steps 5 to 11. Now, observe that in order to generate a permutation distribution under the null hypothesis H_0^{***} (i.e., the classifier is not performing disease recognition and subject identification) we simply need to to shuffle the disease labels in a record-wise fashion. Again, because the record-wise shuffle of the disease labels destroys the associations between labels and features and between labels and subject identities, it turns out that this permutation null will also be centered around 0.5. Furthermore, because a permutation distribution generated by shuffling the labels in a record-wise fashion is actually equivalent to a permutation distribution generated by shuffling the features in a record-wise manner (and to a permutation null where both the labels and the features are independently shuffled in a record-wise manner), it turns out that the spread of the permutation null generated under H_0^{***} will be similar to the spread of the AUC scores computed by the internal for-loop (steps 5 to 11 in Algorithm 3). Hence, it follows that the spread of the permutation null under H_0^{**} will be always smaller than the spread of a permutation null generated under H_0^{***}.

across the distinct subjects (even when the longitudinal feature measurements are independent of each other). Here, we illustrate the application of our permutation tests to synthetic data simulated: (i) in the presence of identity confounding, and absence of disease effect; (ii) in the presence of both identity confounding and disease effect; and (iii) in the absence of both identity confounding and disease effect.

We simulate feature data using a model similar to the one employed by Saeb *et al*[1], except that we generate the data using matrix-normal distributions in order to model the correlation structure across the records and across the features. (The matrix-normal distribution, over the space of matrices with r rows and c columns, is parametrized as,

$$\boldsymbol{W} \sim \text{MN}_{r \times c}(\boldsymbol{M}\,,\, \boldsymbol{\Sigma}\,,\, \boldsymbol{\Psi})\;, \tag{11}$$

where the $r \times c$ matrix $\boldsymbol{M}$ represents the mean of matrix-normal variable $\boldsymbol{W}$; the $r \times r$ matrix $\boldsymbol{\Sigma}$ models the covariance structure across the rows of $\boldsymbol{W}$; and the $c \times c$ matrix $\boldsymbol{\Psi}$ models the covariance structure across the columns of $\boldsymbol{W}$. As an illustration, Figure 1 shows the correlations across rows and columns for data matrices simulated from 4 distinct matrix-normal distributions.)

In our simulations, the feature data matrix from each subject s was generated independently from all other subjects according to the model,

$$\boldsymbol{X}_s = \mu_s \mathbf{1} + a\, y_s \mathbf{1} + b\, \boldsymbol{U}_s + c\, \sigma_s \boldsymbol{V}_s + d\, \boldsymbol{E}_s\;, \tag{12}$$

where a, b, c, and d are scalars; μ_s and σ_s^2 represent, respectively, subject specific mean and variance values (shared by all features); $\mathbf{1}$ corresponds to a matrix with R_s rows and f columns filled with 1s; y_s represents the subject's disease label, assuming the value -1 if the subject is a control and 1 if he/she is a case; and the matrices $\boldsymbol{U}_s$, $\boldsymbol{V}_s$, and $\boldsymbol{E}_s$ are sampled from the matrix-normal distributions,

$$\boldsymbol{U}_s \sim \text{MN}_{R_s \times f}(\mathbf{0}\,,\, \boldsymbol{\Sigma}\,,\, \boldsymbol{I})\;, \tag{13}$$

$$\boldsymbol{V}_s \sim \text{MN}_{R_s \times f}(\mathbf{0}\,,\, \boldsymbol{I}\,,\, \boldsymbol{I})\;, \tag{14}$$

$$\boldsymbol{E}_s \sim \text{MN}_{R_s \times f}(\mathbf{0}\,,\, \boldsymbol{I}\,,\, \boldsymbol{\Psi})\;, \tag{15}$$

where we adopt an autoregressive correlation structure for $\boldsymbol{\Sigma}$ (where each of its elements is given by $\sigma_{ij} = \rho_r^{|i-j|}$, with the fixed scalar ρ_r representing a correlation coefficient), but a simpler correlation structure for $\boldsymbol{\Psi}$ (where the diagonal elements are given by 1, and off-diagonal elements are given by the correlation scalar ρ_f).

The component $\boldsymbol{U}_s$ induces a correlation structure across the records, while $\boldsymbol{E}_s$ generates correlation across the features. The component $\boldsymbol{V}_s$, on the other hand, only contributes white noise, since by adopting identity matrices for the covariances across the records and the features, we have that sampling from $\text{MN}_{R_s \times f}(\mathbf{0}, \boldsymbol{I}, \boldsymbol{I})$ is equivalent to independently sampling each entry of $\boldsymbol{V}_s$ from a standard normal distribution. Also, because $\boldsymbol{U}_s$, $\boldsymbol{V}_s$, and $\boldsymbol{E}_s$ are centered at $\mathbf{0}$, the mean of $\boldsymbol{X}_s$ is given by $\mu_s \mathbf{1} + a\, y_s \mathbf{1}$.

By varying the scalar values μ_s, σ_s, a, b, c, and d in equation (12), we can simulate data under the null and alternative hypothesis for disease recognition and identity confounding. Here, we illustrate the application of our permutation tests in 6 distinct scenarios presented in Figures 2, 3, 4, 5, 6, and 7. Table 1 provides a brief description of the mechanisms giving rise to identity confounding, the hypotheses under which the data was simulated, and the models used to generate the feature data. Our goal is to illustrate how identity confounding can arise for many different reasons, and how the proposed permutation tests are able to detect it.

In all examples, we simulated data for 10 correlated features ($\rho_f = 0.5$), across 20 subjects (13 cases and 7 controls), with the number of records varying from 10 to 20 records per subject, and adopting $\rho_r = 0.95$. We employed 10,000 permutations in the generation of the disease recognition null distribution. The generation of the identity confounding null distribution, on the other hand, was

Example (Figure)	Identity confounding generation mechanism	Hypotheses (H_0 or H_1) consistent with the data	Model used to simulate the feature data, $\boldsymbol{X}_s$
Example 1 (Fig. 2)	serial dependency across records ($\boldsymbol{U}_s$)	disease recog. null (H_0^*), identity conf. alter. (H_1^{**})	$2\,\boldsymbol{U}_s + \boldsymbol{V}_s + 0.5\,\boldsymbol{E}_s$
Example 2 (Fig. 3)	serial dependency across records ($\boldsymbol{U}_s$), and location shifts due to disease label (y_s)	disease recog. alter. (H_1^*), identity conf. alter. (H_1^{**})	$y_s\,\mathbf{1} + 2\,\boldsymbol{U}_s + \boldsymbol{V}_s + 0.5\boldsymbol{E}_s$
Example 3 (Fig. 4)	location shifts due to subject specific mean (μ_s)	disease recog. null (H_0^*), identity conf. alter. (H_1^{**})	$\mu_s\,\mathbf{1} + \boldsymbol{V}_s$
Example 4 (Fig. 5)	location shifts due to disease label (y_s)	disease recog. alter. (H_1^*), identity conf. alter. (H_1^{**})	$y_s\,\mathbf{1} + \boldsymbol{V}_s$
Example 5 (Fig. 6)	scale shifts due to subject specific variances (σ_s^2)	disease recog. null (H_0^*), identity conf. alter. (H_1^{**})	$\sigma_s\,\boldsymbol{V}_s$
Example 6 (Fig. 7)	no identity confounding generation mechanism	disease recog. null (H_0^*), identity conf. null (H_0^{**})	$\boldsymbol{V}_s$

Table 1: Summary of the synthetic data examples. The first column lists the synthetic data examples (and the respective figures where they are presented). The second column describes the mechanisms giving rise to identity confounding in each example. The third describes which disease recognition hypotheses (H_0^* vs H_1^*) and identity confounding hypothesis (H_0^{**} vs H_1^{**}) are consistent with each simulated example. The last column describes the respective models used of simulate the feature data. Further details are provided in the main text.

based on only 1,000 record-wise feature permutations, with 300 subject-wise label permutations per record-wise feature permutation due to computational constraints. (Figure 8, provides a justification for our choice of 300 subject-wise label shufflings. It shows that the sampling variability associated with the adoption of 1,000 record-wise feature permutations overwhelms the small differences in the shape of the identity confounding null achieved by increasing the number of subject-wise label shufflings beyond 300 permutations.)

In our first synthetic data example, we simulated feature data from the model,

$$\boldsymbol{X}_s \,=\, 2\,\boldsymbol{U}_s \,+\, \boldsymbol{V}_s \,+ 0.5\,\boldsymbol{E}_s \;, \tag{16}$$

where identity confounding arises due to the serial correlation across the records induced by the $\boldsymbol{U}_s$ component. Since, the model does not include a disease effect (a was set to 0), we have that the data is consistent with the null hypothesis of the disease recognition permutation test, H_0^*, and with the alternative hypothesis of the identity confounding permutation test, H_1^{**}. Figure 2a shows the ROC curves for data split in a record-wise (brown) and subject-wise (black) fashion. Note that the subject-wise split leads to a much smaller AUC value (0.57) than the record-wise data split (AUC = 0.8). Figure 2b shows boxplots of the feature data across all 20 subjects, with cases and controls shown in orange and green, respectively. In this case, we don't see mean differences between cases and controls, since we simulated featured data centered at 0 (both μ_s and a are set to 0 in the model shown in equation 16). Figure 2c shows the disease recognition null distribution (blue), and the observed AUC value, auc_o, (brown line) for the record-wise data split. The null distribution is centered away from 0.5 (median = 0.77), indicating the presence of identity confounding. The disease recognition permutation p-value (= 0.2984, given by the tail probability of the blue distribution to the right of the brown line), nonetheless, indicates that the classifier is still unable to perform disease recognition, even though the observed AUC value (0.8) is high. This observation suggests that the

high classification performance is entirely due to the classifier's ability to identify subjects. Figure 2d, shows the identity confounding null distribution (red), and the observed value of the $\tilde{auc}^*$ statistic (blue line), which corresponds to the median of the disease recognition null distribution shown in panel c. The identity confounding permutation p-value (computed as the proportion of times that the red distribution was larger than the blue line) is 0, and confirms the presence of identity confounding when the data is split in a record-wise fashion. The grey density represents the analytical approximation for the H_0^{***} null, used in the computation of the the pseudo p-value for detecting identity confounding (given by the area under the grey density to the right of the blue line - which, in this example, is 6×10^{-8}). Figure 2e shows the disease recognition null distribution for the subject-wise data split (blue) and the observed AUC value (black line). Because the subject-wise split (by construction) eliminates the classifier's ability to perform subject identification, this null distribution will always be centered around 0.5. In this example, again, the disease recognition permutation p-value (0.2781) indicates that the random forest classifier is is not performing disease recognition. Figure 2f shows the identity confounding null distribution (red) for the subject-wise data split, and further confirms that this data split avoids identity confounding issues (permutation p-value = 0.719, pseudo p-value = 0.516).

In our second synthetic data example, we simulate data from the model,

$$\boldsymbol{X}_s = y_s \, \boldsymbol{1} + 2 \boldsymbol{U}_s + \boldsymbol{V}_s + 0.5 \, \boldsymbol{E}_s \; , \tag{17}$$

which basically adds a disease effect to the model used in the first example. Hence, the data is now simulated under the alternative hypothesis for disease recognition, H_1^*, and identity confounding, H_1^{**}. Figure 3a shows much higher AUC values for both record-wise (0.97) and subject-wise (0.95) data splits. Figure 3b shows clear mean differences between cases and controls (as one would expect, since we now include a disease effect). Figure 3c shows that, even though identity confounding is playing a role in the random forest's classification performance, when the data is split in a record-wise manner (note the shift away from 0.5 in the blue distribution), the algorithm is, nonetheless, still doing disease recognition (permutation p-value = 0). Figure 3e, shows that the random forest is performing disease recognition with the subject-wise data split, as well (permutation p-value = 0). Figure 3d confirms the presence of identity confounding (permutation p-value = 0, pseudo p-value = 0.0024) with record-wise data split, while Figure 3f confirms it's absence (permutation p-value = 0.318, pseudo p-value = 0.487) with subject-wise split.

In our third example, we illustrate how differences in the mean feature values across the subjects can lead to identity confounding, even when the longitudinal data of each individual has no serial association structure. To this end, we simulate data from the model,

$$\boldsymbol{X}_s = \mu_s \, \boldsymbol{1} + \boldsymbol{V}_s \; , \tag{18}$$

where the feature data of each subject is independent and identically distributed (i.i.d.) according to $\mathrm{N}(\mu_s, 1)$. Note that in this case the data is simulated under the null for disease recognition, H_0^*, and under the alternative for identity confounding, H_1^{**}. (Observe, as well, that this model corresponds to the i.i.d. mixture model for data clustered by subject, discussed in reference[2].) Figure 4b shows accentuated mean differences in feature values across the subjects, and Figures 4c and d illustrate how, even though, the record-wise data split leads to a high AUC value (0.89), the random forest classifier is performing subject identification only (disease recognition permutation p-value = 0.474, identity confounding permutation p-value = 0).

Our fourth example, illustrates how the disease effect can itself induce a certain amount of identity confounding, when the data is split in a record-wise manner. To this end, we simulated data from the model,

$$\boldsymbol{X}_s = y_s \, \boldsymbol{1} + \boldsymbol{V}_s \; , \tag{19}$$

where the feature data from case and control subjects is i.i.d. $\mathrm{N}(1, 1)$ and $\mathrm{N}(-1, 1)$, respectively. Because the disease label contributes to the mean of the feature values, and identity confounding can

arise because of mean differences across the subjects (as illustrated in the previous example), we have that, the data in model (19) is generated under the alternative for disease recognition, H_1^*, as well as, for identity confounding, H_1^{**}. (Observe that, at least for data simulated according to model (12), it is not possible to generate data under the alternative for disease recognition and, concomitantly, under the null for identity confounding.) Figure 5b shows clear mean differences between cases and controls, while Figure 5c shows that the classifier is performing disease recognition with the record-wise data split (permutation p-value = 0). But, more interestingly, it also illustrates that the disease recognition null is slightly shifted away from 0.5 (median = 0.55), showing that the difference in means is enough to generate identity confounding for record-wise data splits. Furthermore, Figure 5d shows that this small shift is statistically significant (identity confounding permutation p-value = 0). Observe, as well, that this example illustrates the case where the pseudo p-value (= 0.162) fails to detect identity confounding, while the permutation p-value does not.

In our fifth example, we illustrate how differences in the variance of the feature values across the subjects can lead to identity confounding, even when the longitudinal data of each individual has no serial association structure. To this end, we simulate data from the model,

$$\boldsymbol{X}_s = \sigma_s \boldsymbol{V}_s , \tag{20}$$

where the feature data of each subject is i.i.d. according to $\text{N}(0, \sigma_s^2)$, and the σ_s^2 values were sampled from a uniform distribution in the interval $(1, 10)$. Note that in this case the data is again simulated under the null for disease recognition, H_0^*, but under the alternative for identity confounding, H_1^{**}. Figure 6b shows clear spread differences between the subjects. Note that although Figure 6c shows that the classifier is not performing disease recognition with the record-wise data split (permutation p-value = 0.3088), it illustrates that the difference in variance is enough to generate identity confounding for record-wise data splits. Furthermore, Figure 6d shows that this small shift is statistically significant (identity confounding permutation p-value = 0). Note, as well, that this example illustrates the case where the pseudo p-value (= 0.069) is marginally significant, while the permutation p-value clearly indicates the presence of identity confounding.

In our sixth example, we consider the null model,

$$\boldsymbol{X}_s = \boldsymbol{V}_s , \tag{21}$$

where all the feature data within and across all participants is i.i.d. $\text{N}(0, 1)$, so that the data is simulated under the null hypothesis for disease recognition, H_0^*, and identity confounding, H_0^{**}. As expected, Figure 7 shows that the random forest classifier is not performing disease recognition or subject identification, no matter how the data is split.

Finally, as a sanity check, we performed a simulation study using 500 distinct data-sets simulated under the null for both disease recognition and identity confounding. Figure 9 reports the results and describes the simulation experiment in more detail. As expected, the distribution of the p-values is approximately uniform for the permutation tests, indicating that the type I error rates are being controlled at the nominal significance levels. The pseudo p-value distributions, on the other hand, are bell shaped and centered around 0.5, indicating that the pseudo p-value approach is conservative at small nominal significance levels (that is, the pseudo p-values tend to be larger than they should be), illustrating, once again, that a small pseudo p-value implies the presence of identity confounding, whereas non-significant pseudo p-values do not imply the absence of digital fingerprints.

3.2 A more detailed analysis of the mPower data

We illustrate the application of our proposed tests to the diagnosis of Parkinson's disease (PD), using iPhone sensor data collected during the first six months of the mPower study[4, 3]. We focus our analyses on data collected from the voice and tapping activity tasks (details about the activity tasks are provided in reference[3]).

Our analyses were based on a matched sample of 11 PD patients and 11 control participants, who provided at least 100 records over the 6 month period. All case and control participants were males, and were first matched by age (using exact matching), and then by education level (using nearest neighbor matching[9]) whenever there were multiple cases with the same age of a control, or vice-versa. (In the event that ties persisted after the application of this second matching criterion, we randomly selected one participant to perform the matching.) We trained separate random forest classifiers for the voice and tapping data, based on 13 voice features and 41 tapping features proposed in the literature[10, 11].

Due to the small number of subjects, in order to maintain balance in the age distributions of cases and controls in the training and test sets, we performed an age balanced version of the subject-wise data split (where whenever the training set contained a case of a given age, then the test set contained the matched control of the same age, and vice versa). For the record-wise data split, on the other hand, we simply randomly selected the records. (Note that because each subject has a large number of records (at least one hundred), the distribution of the ages tends to be well balanced between cases and controls in both the training and test sets.)

Figure 10 shows the results for the voice data. From panel a we observe a massive amount of identity confounding for the record-wise split data, with the disease recognition null distribution (blue) concentrated at very high AUC values (median AUC = 0.954). Both the identity confounding permutation p-value and the pseudo p-value are 0 in this example. The disease recognition p-value (= 0.5843), on the other hand, shows that the random forest classifier is not performing disease recognition, even though the observed AUC (brown line) is 0.952. Panel b reports the results for the subject-wise data split, and show that, once again, the classifier is not learning about the disease labels (disease recognition p-value = 0.7243).

In order to assess the robustness of these results with respect to the training/test data split, we show in panel c the disease recognition null distributions (blue boxplots) for 30 distinct record-wise data splits, as well as, the respective AUC values (brown dots), and the distribution of the disease recognition permutation p-values across the 30 data splits (brown boxplot). The results show that in none of the 30 data splits we observed a permutation p-value smaller than 0.05 (dashed red line), showing that the very high AUC values (brown dots) achieved by the classifier are due largely to the classifier's ability to identify subjects. Finally, panel d reports the results for the subject-wise data split, confirming that the classifier is indeed unable to perform disease recognition.

Figure 11 shows the results for the tapping data. Again from panel a we observe high levels of identity confounding for the record-wise data split (identity confounding permutation p-value and the pseudo p-value are again 0), but contrary to the voice data, the random forest classifier is now able to perform disease recognition with the tapping features (disease recognition p-value = 0.014). Panel b, shows that the classifier trained with the subject-wise data split is still able to perform disease recognition, although the statistical significance is weaker (permutation p-value = 0.055). Panel c shows that the results presented in panel a are robust across distinct record-wise data splits. Panel d assesses the robustness of the findings observed in panel b, and shows that results tend to be more variable and less significant in this case when compared to the record-wise data splits (note how the distribution of the disease recognition p-values have a larger spread than in the record-wise case). We point out, nonetheless, that this larger variability is likely due to the very small number of subjects and the heterogeneous nature of the data, and suggests that a certain amount of model under-fitting is going on in the tapping data.

In order to assess whether a larger number of subjects would improve the power to perform disease recognition (especially when adopting the subject-wise data split strategy) we repeated our analysis using a larger number of subjects (who, nonetheless, provided a smaller number of records). Specifically, for the voice data we analyzed two additional (age matched and male) cohorts containing 42 subjects (who provided at least 50 records), and 240 subjects (who contributed at least 10 records), whereas for the tapping data we analyzed two additional cohorts containing 48 and 290 subjects (who contributed at least 50 and 10 records, respectively). As before, we ran the

analyses on 30 distinct data splits, with half of the subjects and (approximately) half of the records assigned to training set in the subject-wise and record-wise data splits, respectively.

Figure 12 reports the results for the voice data. Panels a, b, and c show the disease recognition null distributions and observed AUC values for the record-wise data splits across increasing numbers of subjects. In all 3 panels, the disease recognition null distributions are concentrated around very high AUC values, suggesting that identity confounding is playing an important role in all three cohorts. It is interesting to note that the AUC values tended to decrease, as the amount of data used to train the random forests increased. This drop in AUC is, nevertheless, not surprising, given that the classifier is mostly performing subject identification and that a larger number of subjects makes it harder to identify the individual subjects. Observe, as well, that the random forest only started to detect the disease signal in panel c, where a larger amount of data was available to train the classifier (note how the brown dots started to approach the upper tail of the blue boxplots). Panels d, e, and f, show analogous results for the subject-wise data splits. Note that, as expected, the disease recognition null distributions were always centered around 0.5, and their spread tended to decrease as the number of subjects increased. Observe, nonetheless, that the random forest was unable to perform disease recognition even when the training set contained data from 120 subjects (panel f). Panel g shows the distributions of the disease recognition permutation p-values, across the 30 random data splits shown in panels a to f.

Figure 13 shows the results for the tapping task. Overall, we observe the same patterns as for the voice data, except that tapping features seem to be less prone to perform subject identification (comparison of panels a, b, and c on Figures 13 and 12 show that both the observed AUC values and disease recognition null distributions tend to be located at lower AUC values for the tapping data than for the voice data), and are better able to perform disease recognition in both record-wise and subject-wise data splits (compare Figures 13g and 12g).

Our analyses of the voice and tapping data, collected by the mPower study, showed undeniable evidence of a high degree of identity confounding for classifiers built using record-wise data splits. Although it is true that our results also showed evidence of model under-fitting for classifiers trained with subject-wise data splits, we point out that model under-fitting can be ameliorated by simply increasing the number of subjects used in the analyses. (The rationale is that, as the number of subjects used to train the classifier increases, the chance that the training set is missing a critical part of the pattern that relates features to disease labels decreases, so that the classifier has a better chance to generalize to new unseen cases, even when the data is fairly heterogeneous). But, most importantly, our data does not support the hypothesis raised by the first author of Little *et al*[2] that model under-fitting (alone), rather than identity confounding, could explain the discrepancy in classification performance between the record-wise and subject-wise strategies.

Interestingly, the voice data seems to require a much larger number of subjects in order to perform disease recognition. The fact that the random forest classifier was unable to perform disease recognition with the subject-wise data splits, and only started to detect the disease signal with record-wise data splits when the training set contained over 100 subjects, combined with the fact that the voice-based classifier achieved higher AUC values than the tapping-based one in the record-wise data splits, suggests that our voice features are more vulnerable to identity confounding and less suited to perform disease recognition, when compared to the tapping features. A possible explanation, is that voice features are intrinsically better able to capture personal and physiological characteristic of a subject compared to a “game” based tapping feature. An alternative explanation, is that the weaker disease recognition signal might be due to suboptimal processing and cleaning of the voice raw data. To see why, note that the digital fingerprint of a subject may arise not only from biological characteristics, such as the pitch of a subject’s voice, but also from non-biological/environmental artifacts, such as the amount of background noise and the distance of the subject’s mouth to the phone’s microphone. These non-biological artifacts can certainly contribute to identify subjects when the voice tasks are performed under different conditions across the subjects, but under consistent conditions within each subject. (Note that while this issue can be somewhat controlled in laboratory

experiments, this is not generally the case in mobile health studies, such as mPower, that are conducted at home under uncontrolled conditions.) This problem is likely exacerbated by the use of highly sensitive sensors, capable of detecting quite subtle variations in sound. The tapping task, on the other hand, seems to provide less room for environmental artifacts.

Finally, while all analyses of the mPower data presented so far were based in data splits assigning 50% of the data to training set and 50% to the test set (denoted, for short, as the 50/50 split), Figures 14 and 15 compare the results based on 50/50 record-wise data splits to 90/10 splits (i.e., record-wise data splits assigning 90% of the records to training set and 10% to the test set) for the tapping and voice data, respectively. In all panels, the blue boxplots show the permutation null distributions, the brown dots show the observed AUC score, and the blue and brown horizontal lines show the median (across all 30 distinct data splits) of the permutation null distributions and observed AUC scores, respectively. Comparison of panels a versus b, c versus d, e versus f, show that for both tapping (Figure 14) and voice (Figure 15) datasets the 90/10 split allowed the classifiers to better identify the subjects. (Note that in all comparisons the horizontal blue lines are higher for the 90/10 splits in comparison with the 50/50 splits. Similarly, the horizontal brown lines were also higher for the 90/10 splits relative to the 50/50 splits.) On the other hand, because the test sets were considerably smaller for the 90/10 splits, the spreads to the permutation null distributions tended to be larger, and the results also tended to be more variable for the 90/10 splits (in the sense that the blue boxplots were more widespread around the blue horizontal line, and the brown dots were more widespread around the brown horizontal line). Furthermore, for the tapping data (Figure 14), note how the brown dots tended to be further away at the tail of the permutation nulls for the 50/50 splits in comparison with the 90/10 splits. This suggests that, as expected, the ability of the classifiers to perform disease recognition tended to decrease in the 90/10 splits. (For the voice data, on the other hand, the results tended to be less clear cut since the classifiers were already having difficulty to learn the disease signal with the 50/50 split.)

References

[1] Saeb S, Lonini L, Jayaraman A, Mohr DC, Kording KP. The need to approximate the use-case in clinical machine learning. GigaScience. 2017; 6: 1-9. doi:doi:10.1093/gigascience/gix019

[2] Little M, Varoquaux G, Saeb S, Lonini L, Jayaraman A, Mohr DC, Kording KP. Using and understanding cross-validation strategies. Perspectives on Saeb et al. GigaScience. 2017; 6: 1-9. doi:10.1093/gigascience/gix020

[3] Bot BM et al. The mPower study, Parkinson disease mobile data collected using ResearchKit. Scientific Data, 2016; 3:160011 doi:10.1038/sdata.2016.11

[4] Trister AD, Dorsey ER, Friend SH. Smartphones as new tools in the management and understanding of Parkinsons disease. npj Parkinson's Disease, 2016; 16006.

[5] Bamber D. The area above the ordinal dominance graph and the area below the receiver operating characteristic graph. Journal of Mathematical Psychology. 1975; 12: 387-415.

[6] Mason SL, Graham NE. Areas beneath the relative operating characteristics (ROC) and relative operating levels (ROL) curves: statistical significance and interpretation. Quarterly Journal of the Royal Meteorological Society. 2002. 128: 2145-2166.

[7] Breiman L. Random forests. Machine Learning. 2001; 45: 5-32.

[8] Liaw A, Wiener M. Classification and regression by randomForest. R News. 2002; 2: 18-22.

[9] Ho D, Imai K, King G, Stuart EA. MatchIt: Non-parametric preprocessing for parametric causal inference. Journal of Statistical Software, 2011; 42: 1-28.

[10] Tsanas A, Little MA, McSharry PE, Ramig LO. Nonlinear speech analysis algorithms mapped to a standard metric achieve clinically useful quantification of average Parkinson's disease symptom severity. J R Soc Interface, 2011; 8: 842-855.

[11] Arora, S et al. Detecting and monitoring the symptoms of Parkinsons disease using smartphones: A pilot study. Parkinsonism Relat. Disord, 2015; 21: 650653.

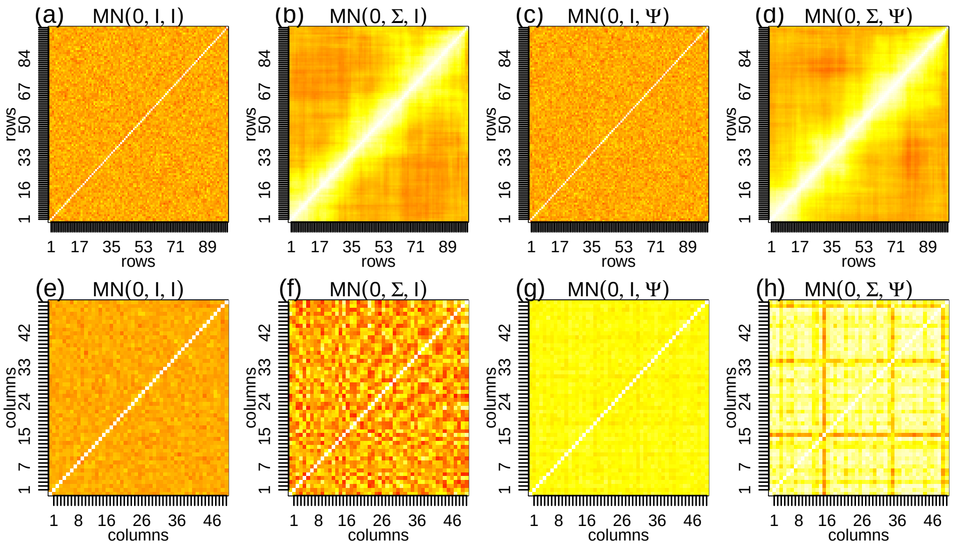

Figure 1: Examples of correlation structures generated with the matrix-normal distribution. The top and bottom panels show, respectively, the correlation structure across the rows and columns, for data matrices of dimension 100×50 sampled from 4 distinct matrix-normal distributions (listed above the heatmaps). Lighter colors represent stronger correlation values. In all examples, we adopted $\rho_r = 0.95$, and $\rho_f = 0.5$ (see Section 3.1). Panels a and e illustrate the case where the data was generated without correlation structures across the rows and columns. Panels b and f depict the case with serial correlation structure across the rows, but no correlation across the columns. Panels c and g, illustrate the case with correlation across the columns, but not across the rows. Finally, panels d and h, depict the case with correlation structure across both rows and columns.

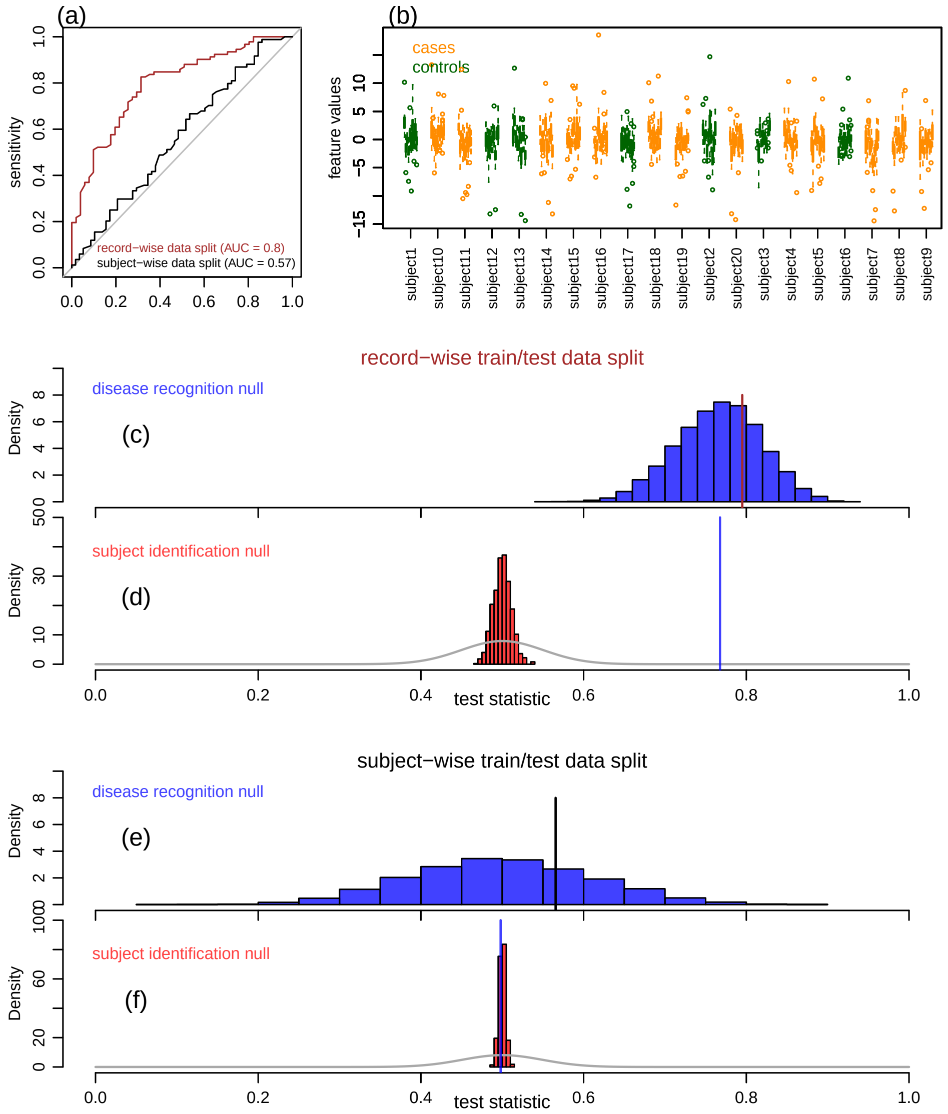

Figure 2: Synthetic data example 1, where the feature data was simulated from model $\boldsymbol{X}_s = 2\,\boldsymbol{U}_s + \boldsymbol{V}_s + 0.5\,\boldsymbol{E}_s$, under the null hypothesis for disease recognition, H_0^* and the alternative hypothesis for identity confounding, H_1^{**}. Panel a shows the ROC curves and AUC values for data split in a record-wise (brown) and subject-wise (black) fashion. Panel b shows boxplots of the feature data across all subjects, with cases and controls shown in orange and green, respectively. Panels c and d show, respectively, the disease recognition and the identity confounding permutation null distributions (blue and red histograms) for the record-wise data split. The brown line in panel c corresponds to the observed AUC value, while the blue line and grey curve in panel d show, respectively, the median of the blue histogram in panel c and the density of the normal distribution used for the computation of the pseudo p-value. Panels e and f show the analogous objects for the subject-wise data split.

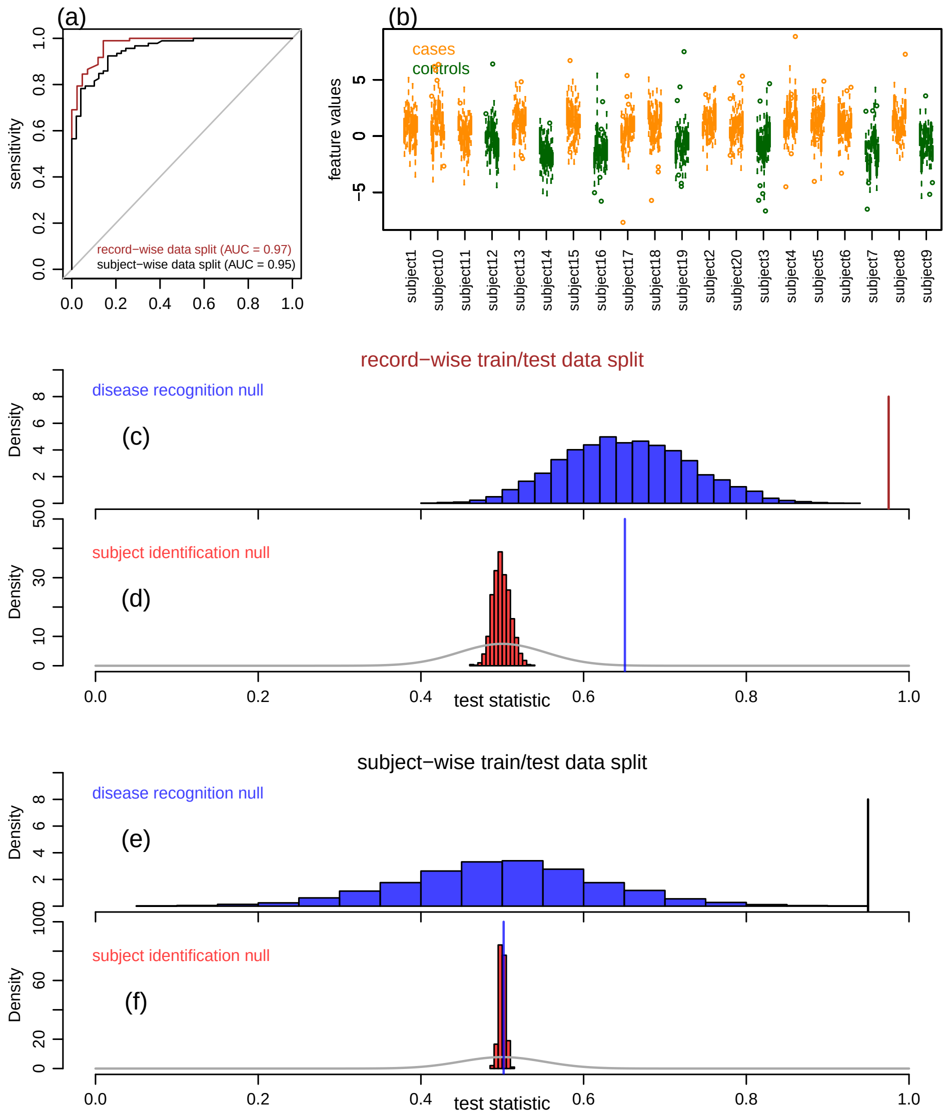

Figure 3: Synthetic data example 2, where the feature data is simulated from model $\boldsymbol{X}_s = y_s \boldsymbol{1} + 2\boldsymbol{U}_s + \boldsymbol{V}_s + 0.5\,\boldsymbol{E}_s$, under the alternative hypothesis for disease recognition, H_1^*, and the alternative hypothesis for identity confounding, H_1^{**}. Panel a shows the ROC curves and AUC values for data split in a record-wise (brown) and subject-wise (black) fashion. Panel b shows boxplots of the feature data across all subjects, with cases and controls shown in orange and green, respectively. Panels c and d show, respectively, the disease recognition and the identity confounding permutation null distributions (blue and red histograms) for the record-wise data split. The brown line in panel c corresponds to the observed AUC value, while the blue line and grey curve in panel d show, respectively, the median of the blue histogram in panel c and the density of the normal distribution used for the computation of the pseudo p-value. Panels e and f show the analogous objects for the subject-wise data split.

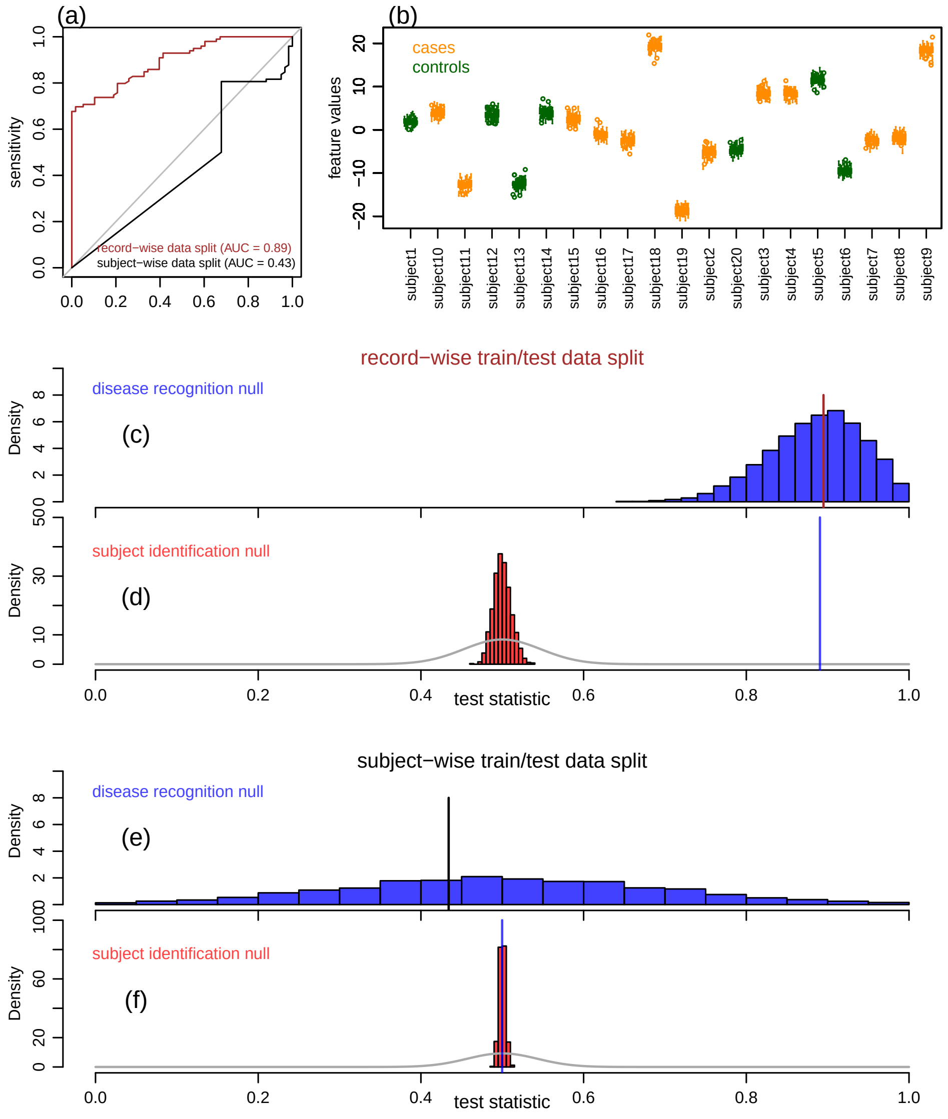

Figure 4: Synthetic data example 3, where the feature data is simulated from model $\boldsymbol{X}_s = \mu_s \mathbf{1} + \boldsymbol{V}_s$, under the null hypothesis for disease recognition, H_0^*, and the alternative hypothesis for identity confounding, H_1^{**}. Panel a shows the ROC curves and AUC values for data split in a record-wise (brown) and subject-wise (black) fashion. Panel b shows boxplots of the feature data across all subjects, with cases and controls shown in orange and green, respectively. Panels c and d show, respectively, the disease recognition and the identity confounding permutation null distributions (blue and red histograms) for the record-wise data split. The brown line in panel c corresponds to the observed AUC value, while the blue line and grey curve in panel d show, respectively, the median of the blue histogram in panel c and the density of the normal distribution used for the computation of the pseudo p-value. Panels e and f show the analogous objects for the subject-wise data split.

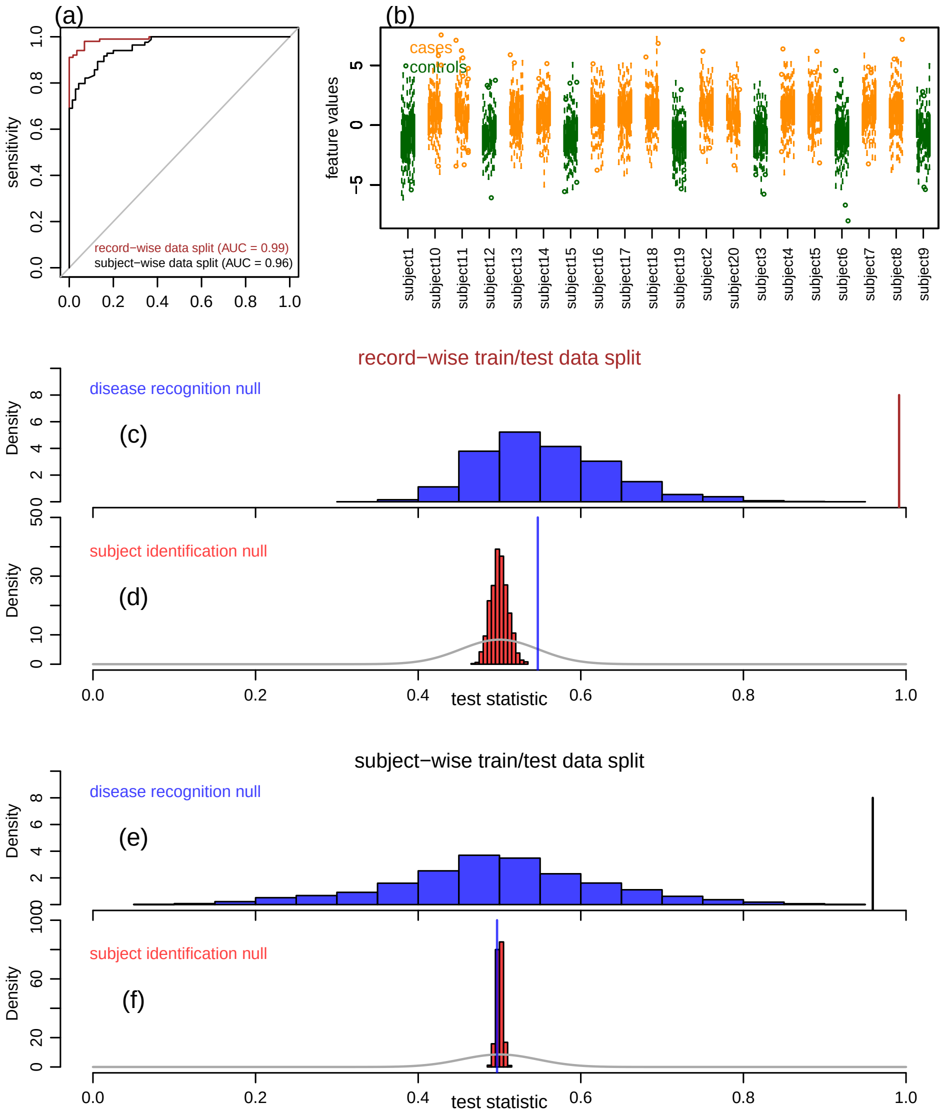

Figure 5: Synthetic data example 4, where the feature data is simulated from model $\boldsymbol{X}_s = y_s\,\mathbf{1} + \boldsymbol{V}_s$, under the alternative hypothesis for disease recognition, H_1^*, and the alternative hypothesis for identity confounding, H_1^{**}. Panel a shows the ROC curves and AUC values for data split in a record-wise (brown) and subject-wise (black) fashion. Panel b shows boxplots of the feature data across all subjects, with cases and controls shown in orange and green, respectively. Panels c and d show, respectively, the disease recognition and the identity confounding permutation null distributions (blue and red histograms) for the record-wise data split. The brown line in panel c corresponds to the observed AUC value, while the blue line and grey curve in panel d show, respectively, the median of the blue histogram in panel c and the density of the normal distribution used for the computation of the pseudo p-value. Panels e and f show the analogous objects for the subject-wise data split.

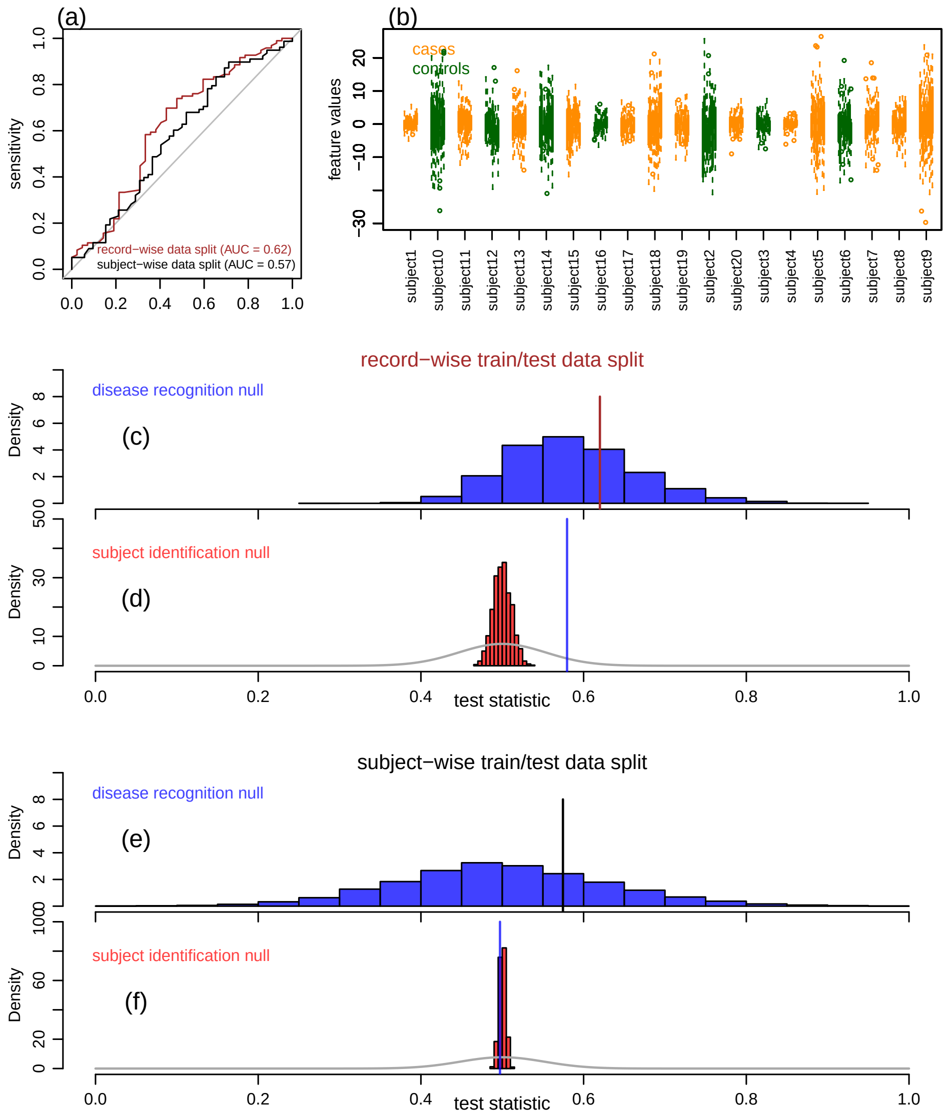

Figure 6: Synthetic data example 5, where the feature data is simulated from model $\boldsymbol{X}_s = \sigma_s \boldsymbol{V}_s$, under the null hypotheses for disease recognition, H_0^*, and identity confounding, and the alternative hypothesis for identity confounding, H_1^{**}. Panel a shows the ROC curves and AUC values for data split in a record-wise (brown) and subject-wise (black) fashion. Panel b shows boxplots of the feature data across all subjects, with cases and controls shown in orange and green, respectively. Panels c and d show, respectively, the disease recognition and the identity confounding permutation null distributions (blue and red histograms) for the record-wise data split. The brown line in panel c corresponds to the observed AUC value, while the blue line and grey curve in panel d show, respectively, the median of the blue histogram in panel c and the density of the normal distribution used for the computation of the pseudo p-value. Panels e and f show the analogous objects for the subject-wise data split.

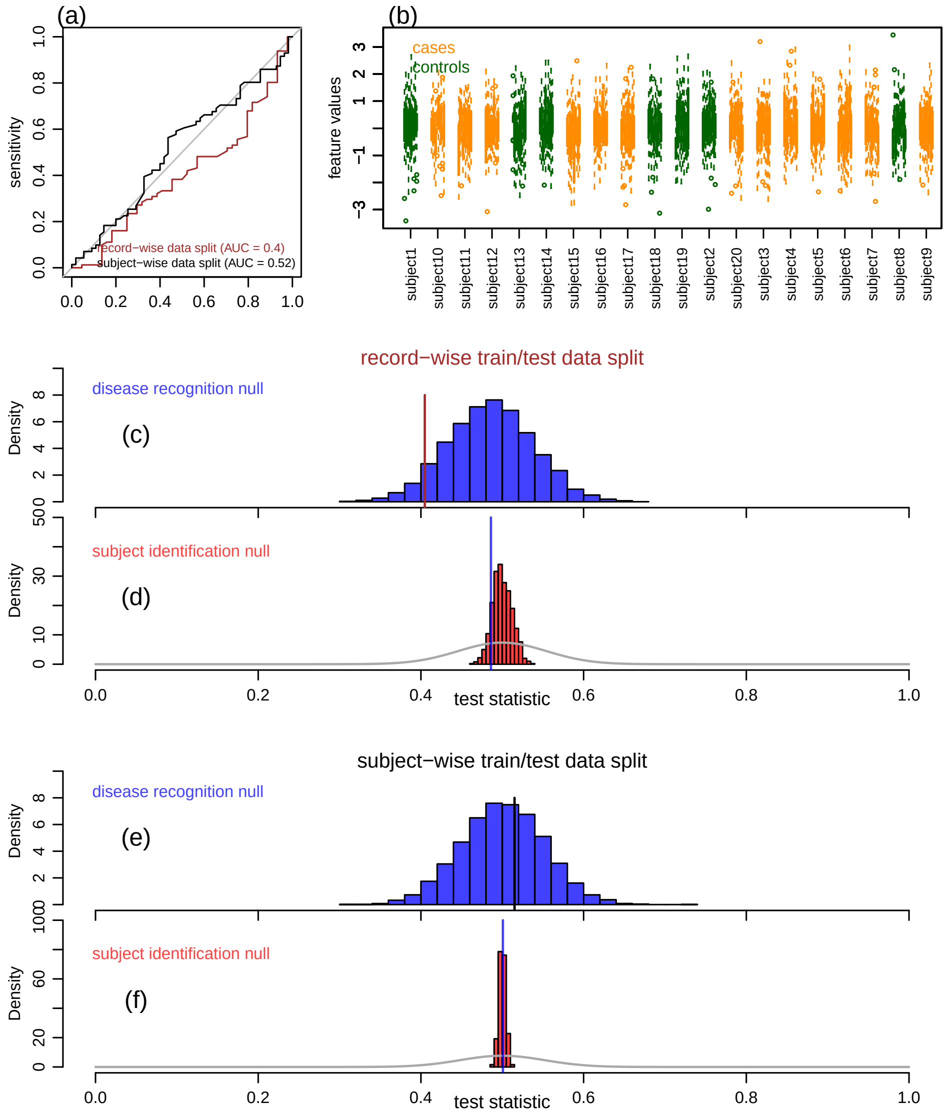

Figure 7: Synthetic data example 6, where the feature data is simulated from model $\boldsymbol{X}_s = \boldsymbol{V}_s$, under the null hypotheses for disease recognition, H_0^*, and identity confounding, H_0^{**}. Panel a shows the ROC curves and AUC values for data split in a record-wise (brown) and subject-wise (black) fashion. Panel b shows boxplots of the feature data across all subjects, with cases and controls shown in orange and green, respectively. Panels c and d show, respectively, the disease recognition and the identity confounding permutation null distributions (blue and red histograms) for the record-wise data split. The brown line in panel c corresponds to the observed AUC value, while the blue line and grey curve in panel d show, respectively, the median of the blue histogram in panel c and the density of the normal distribution used for the computation of the pseudo p-value. Panels e and f show the analogous objects for the subject-wise data split.

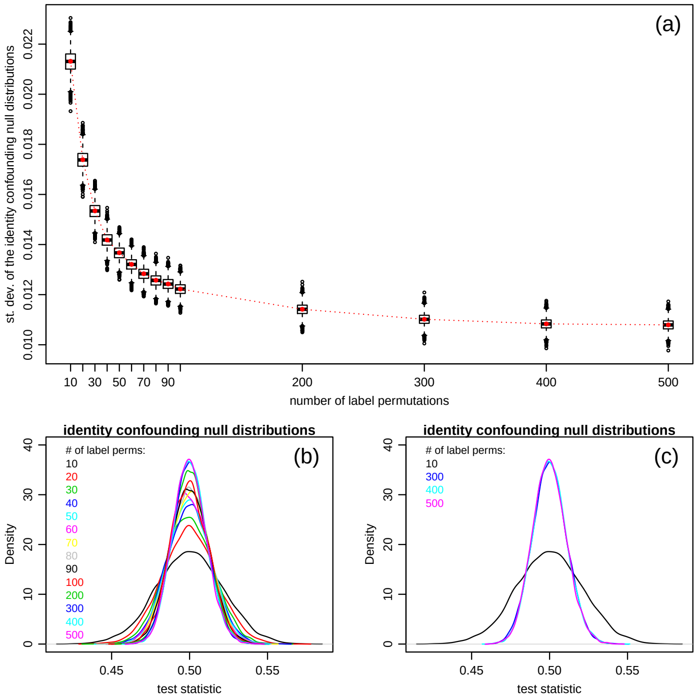

Figure 8: Determination of a sufficient number of subject-wise label permutations in the generation of the identity confounding null distribution. As described in Algorithm 2, in the main text, the generation of the identity confounding null distribution requires the computation of the disease recognition null distribution (which is based on p_l subject-wise label shufflings), for each one of the p record-wise feature data permutations, so that the total number of computations is given by $p \times p_l$. Note, nonetheless, that because the test statistic ($\tilde{m}^*$) corresponds to the median of a performance metric (m^*), we don't need to use a large number of label permutations, p_l (since it is reasonable to expect that the median of the disease recognition null distribution, computed with, say, 1,000 subject-wise label permutations should be close to the median computed with 10,000 permutations). This rationale suggests, that we might be able to determine a sufficient number of label permutations, by computing the identity confounding null distribution using increasing p_l values, and inspecting when the null distribution "stabilizes". Panel a shows the standard deviation of the identity confounding null distribution (red dots) computed with $p = 10,000$ record-wise feature permutations for p_l varying from 10 to 500 subject-wise label permutations. The black boxplots show the distributions of the standard deviations across 10,000 sub-samples of size 1,000 of the respective identity confounding null distributions, and provide estimates of the amount of sampling variability we should expect when we adopt 1,000 record-wise feature permutations, rather than 10,000. The panel shows that, while the standard deviation estimates based on $p = 10,000$ permutations (red dots) are still monotonically decreasing as p_l increases, the sampling variability that we see when we adopt $p = 1,000$ by far overwhelms the small difference in standard deviation that is achieved by increasing p_l from 300 to 400 (or 500). Similarly, panel b shows that the identity confounding null distribution tends to stabilize as the number of label permutations increases, and panel c shows that the null distributions are already very similar when we adopt $p_l = \{300, 400, 500\}$. All the results were based on data simulated according to synthetic example 1 (equation 16), in the main text.

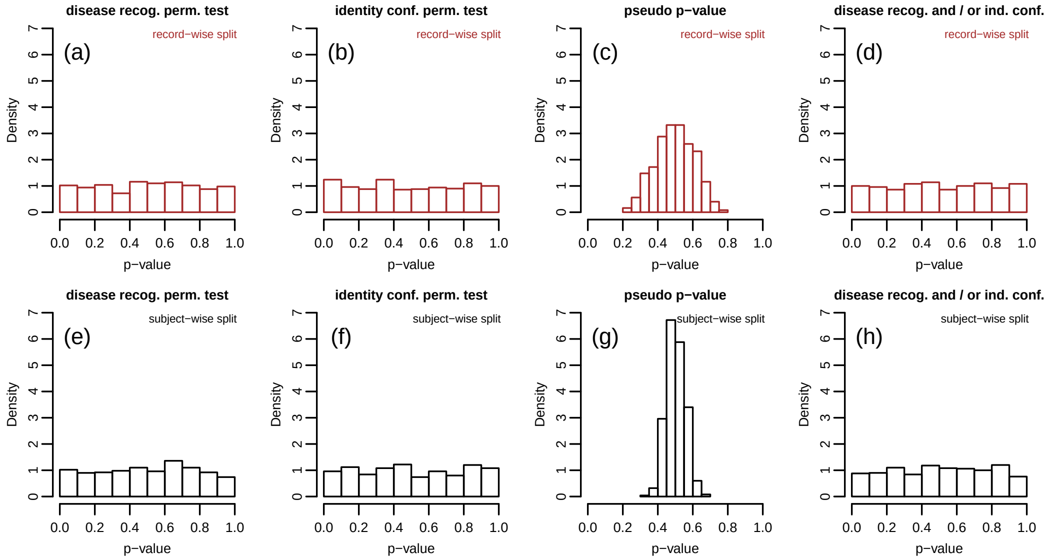

Figure 9: Assessing type I error rate control with a simulation study. We simulated 500 distinct data-sets under the null hypothesis of both the disease recognition and the identity confounding tests. For each synthetic data-set, the feature data of each subject was independently simulated according to the model, $\boldsymbol{X}_s = c\,\boldsymbol{V}_s + d\,\boldsymbol{E}_s$, in order to allow for correlation across the features (see main text for further details). Each simulated data-set was generated with a unique combination of simulation parameters, with the scalars c and d varying from 0.1 to 2, the number of cases and controls varying from 5 to 10, and the number of records per participant varying from 10 to 20. In order to select parameter values spread as uniformly as possible over the entire parameter ranges, we adopted a Latin hypercube space filling design in the determination of the parameter values used to generate each data-set. Due to computational constraints, the permutation tests were based on 100 permutations. The top panels show the results from tests applied to data-sets split in a the record-wise fashion, while the bottom panels report the results based on subject-wise data splits. Panels a and e show the p-value distributions from the disease recognition permutation test, while panels b and f show the p-value distributions from the identity confounding permutation test. As expected, the distributions are approximately uniform showing that the type I error rate of the permutation tests are being controlled at the nominal significance levels. Panels c and g show the distributions of the pseudo p-values. As expected, the distributions are bell-shaped since a central limit theorem for the sample median of a random variable is kicking in (note that because the pseudo p-value is computed using the median of the AUC metric, and the tail probability computed with the median of a sample of test statistics is equal to the median of the tail probabilities computed for each test statistic in the sample, that is, $1 - \Phi((\text{median}_j\{\text{auc}_j^*\} - 0.5)/\phi) = \text{median}_j\{1 - \Phi((\text{auc}_j^* - 0.5)/\phi)\}$, we have that the pseudo p-value distributions actually correspond to the distributions of the sample median of disease recognition p-value distributions). Finally, panels d and h, report the p-value distributions from the analytical test for the presence of disease recognition and/or identity confounding (H_0^{***}) calculated according to expression (8), in the main text. As expected, the p-value distributions are also approximately uniform.

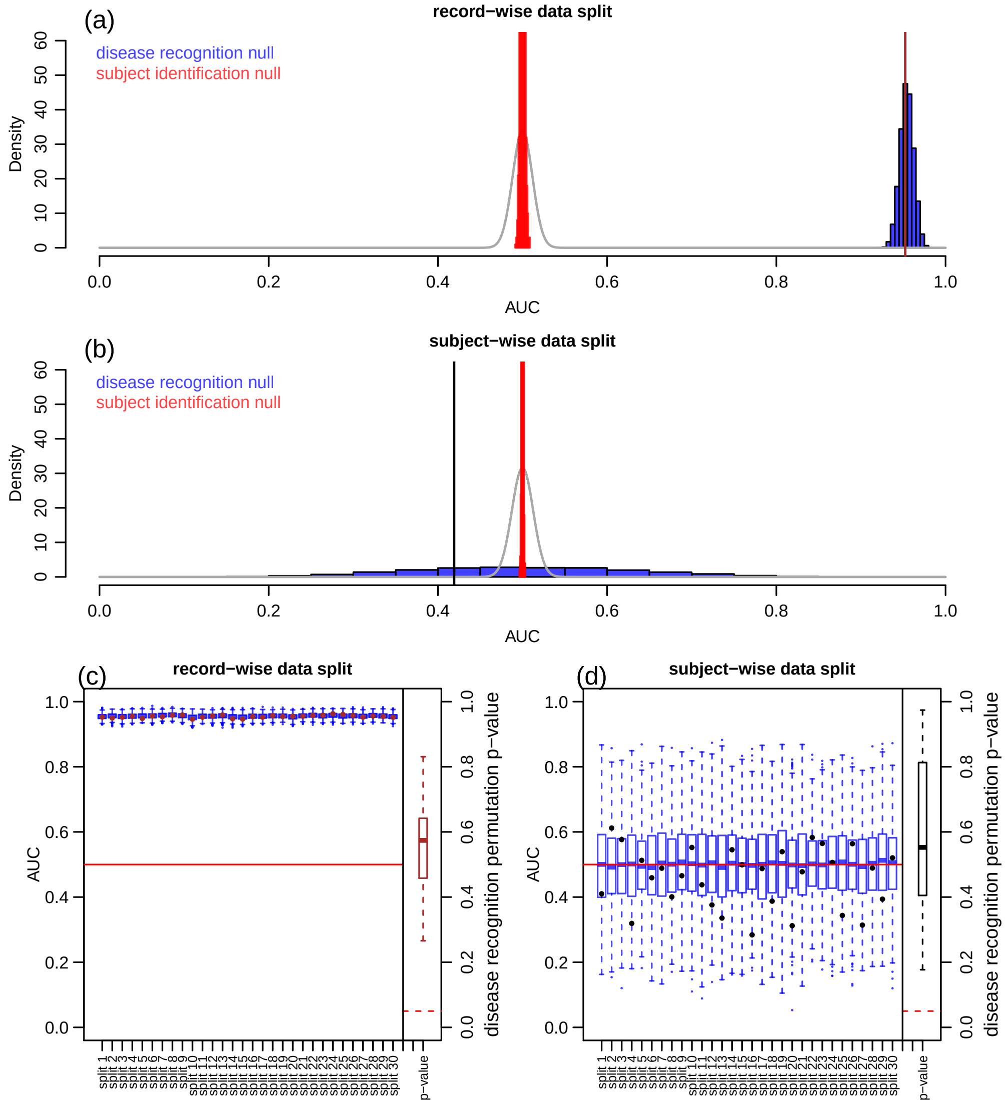

Figure 10: Voice data example. Panel a shows the disease recognition permutation null (blue) the observed AUC value (brown line), the identity confounding permutation null distribution (red), and the density of the analytical null distribution used for the computation of the pseudo p-values, for the record-wise data split. Panel b shows the analogous objects for the subject-wise data split. Panel c reports the disease recognition null distributions (blue boxplots) for 30 distinct record-wise data splits, as well as, the observed AUC values (brown dots), and the distribution of the disease recognition permutation p-values across the 30 data splits (brown boxplot). Panel d shows the analogous objects for the subject-wise data split. The disease recognition nulls was generated with 10,000 permutations in panels a and b, and 1,000 permutations in panels c and d. The identity confounding nulls were generated with 1,000 feature permutations (and 300 label permutations per feature permutation).

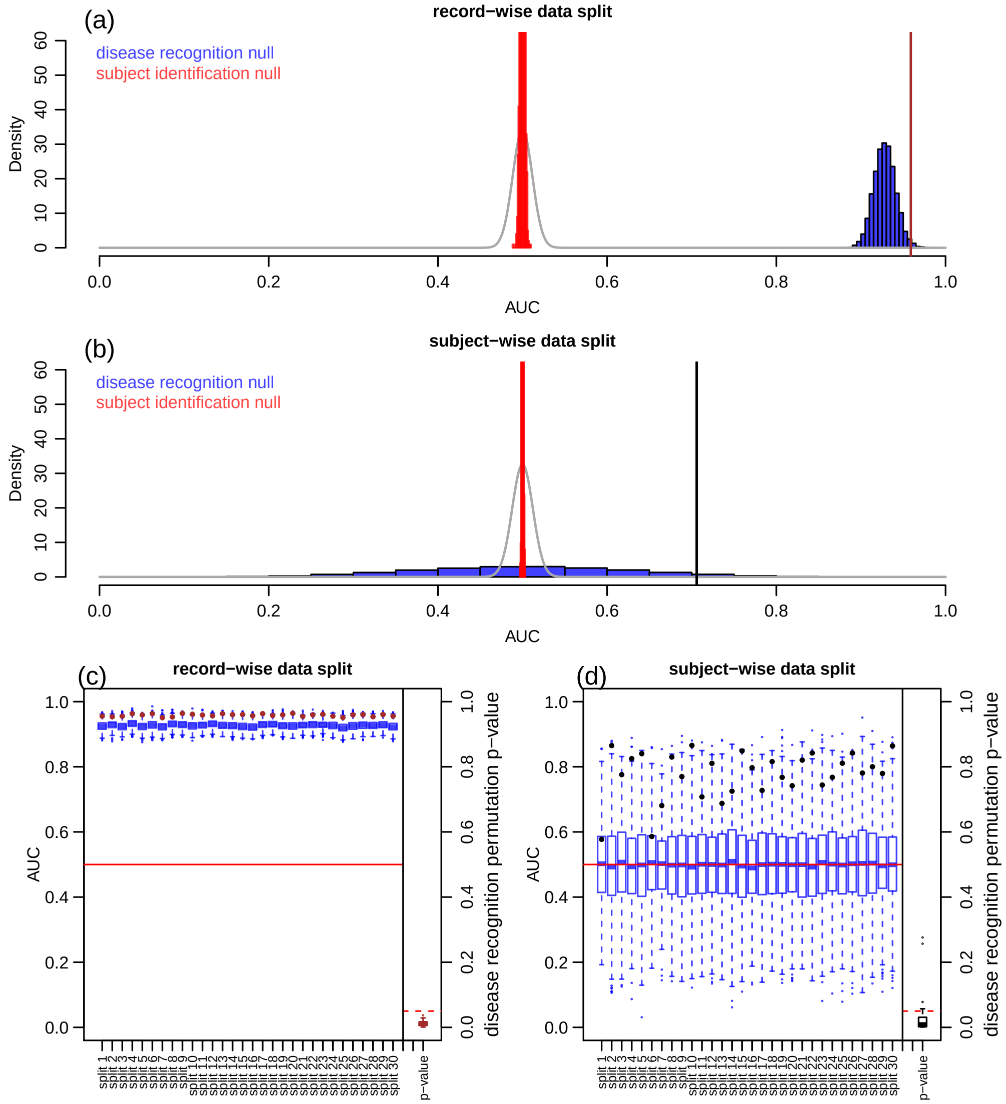

Figure 11: Tapping data example. Panel a shows the disease recognition permutation null (blue) the observed AUC value (brown line), the identity confounding permutation null distribution (red), and the density of the analytical null distribution used for the computation of the pseudo p-values, for the record-wise data split. Panel b shows the analogous objects for the subject-wise data split. Panel c reports the disease recognition null distributions (blue boxplots) for 30 distinct record-wise data splits, as well as, the observed AUC values (brown dots), and the distribution of the disease recognition permutation p-values across the 30 data splits (black boxplot). Panel d shows the analogous objects for the subject-wise data split. The disease recognition nulls was generated with 10,000 permutations in panels a and b, and 1,000 permutations in panels c and d. The identity confounding nulls were generated with 1,000 feature permutations (and 300 label permutations per feature permutation).

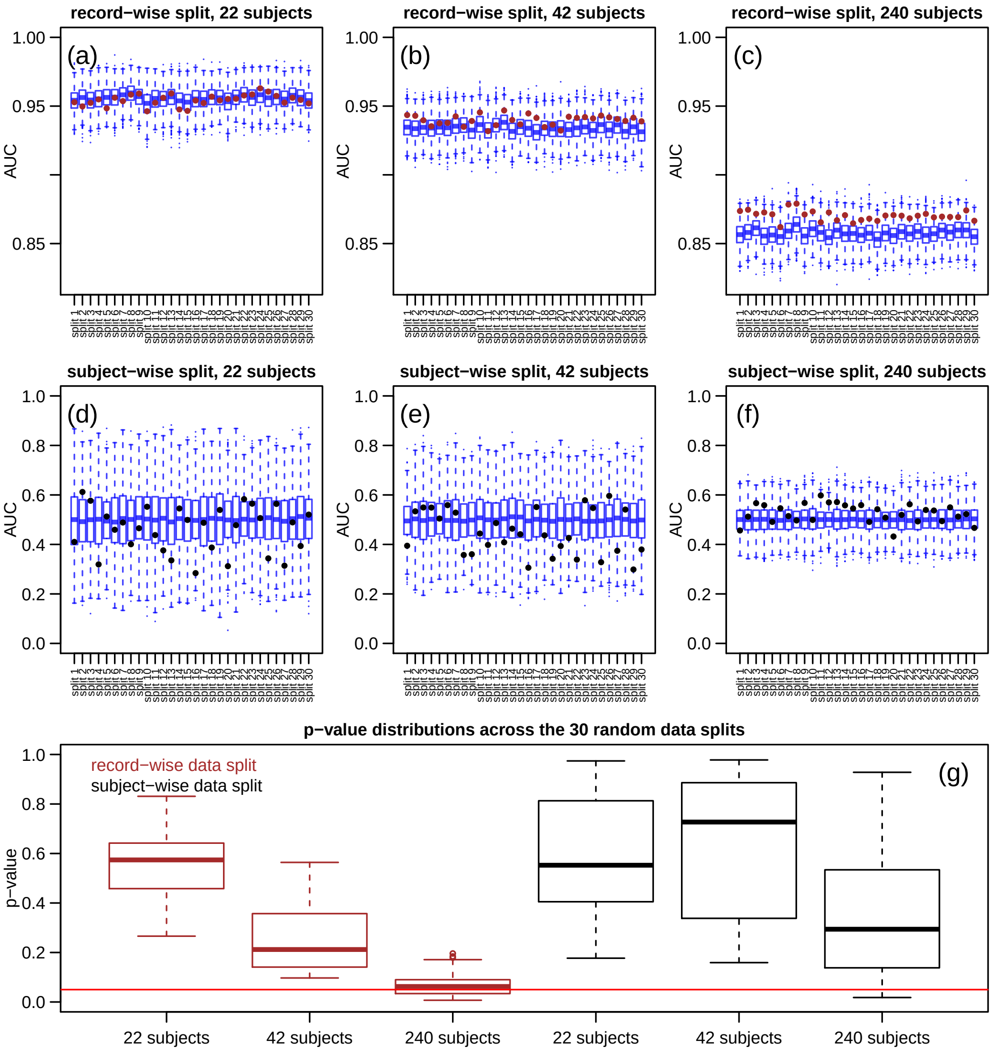

Figure 12: Comparison of the disease recognition performance in the voice data, across increasing number of subjects. Panels a, b, and c, show the disease recognition null distributions (blue boxplots), across 30 distinct record-wise data splits, for classifiers trained and evaluated using, respectively, 22 subjects (with at least 100 records per subject), 42 subjects (with at least 50 records per subject), and 240 subjects (with at least 10 records). The brown dots represent the observed AUC values. Observe the decrease in AUC, as the number of subjects increase. Panels d, e, and f, show the analogous results for 30 distinct subject-wise data splits (with black dots representing the observed AUC values). Note the decrease in the spread of the disease recognition null distributions, as the number of subjects increases. (Observe, as well, the different y-axis scales of panels a, b, and c, when compared to panels d, e, and f.) Panel g shows the distributions of the disease recognition permutation p-values, across the 30 random data splits shown in panels a to f. Note that in none of the data splits the permutation p-value was smaller than 0.05 (red line), showing that the classifier is unable to perform disease recognition with the adopted voice features.

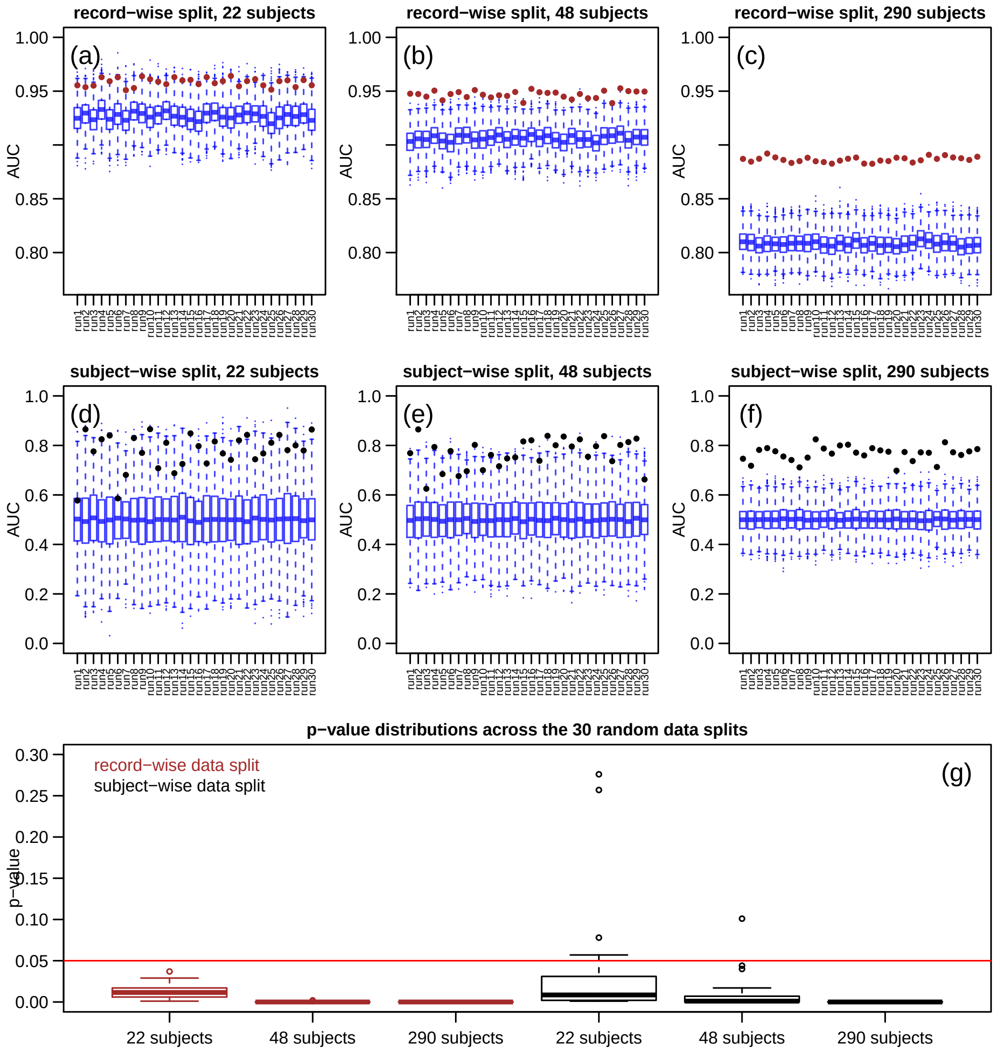

Figure 13: Comparison of the disease recognition performance in the tapping data, across increasing number of subjects. Panels a, b, and c, show the disease recognition null distributions, across 30 distinct record-wise data splits, for classifiers built with, 22 subjects (with at least 100 records per subject), 48 subjects (with at least 50 records per subject), and 290 subjects (with at least 10 records), respectively. The brown dots represent the observed AUC values. Observe the decrease in AUC, as the number of subjects increase. Panels d, e, and f, show the analogous results for 30 distinct subject-wise data splits (with black dots representing the observed AUC values). Note the decrease in the spread of the disease recognition null distributions, as the number of subjects increases. Panel g shows the distributions of the disease recognition permutation p-values, across the 30 random data splits shown in panels a to f. Note how the p-values tend to decrease, as the number of subjects increase. In particular, the accentuated improvement in power for the subject-wise split strategy (black boxplots), illustrates how the increase in the number of subjects reduced to a great extent the model under-fitting issue observed in panel d.

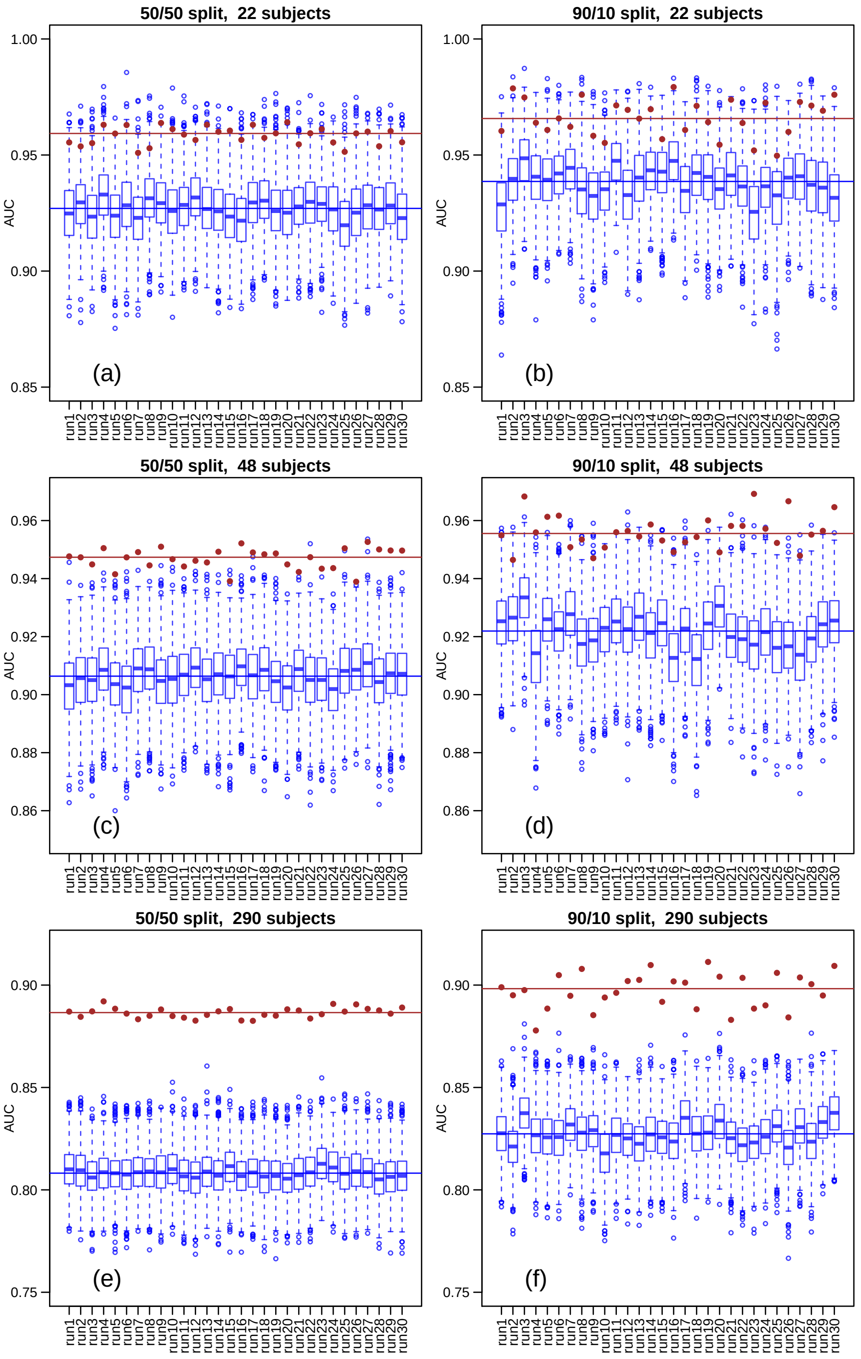

Figure 14: Comparison of record-wise data splits assigning 50% of the records to the training set and 50% to the text set (50/50 split) versus record-wise data splits assigning 90% of the records to the training set and 10% to the text set (90/10 split) for the mPower tapping data across 30 distinct data splits. In all panels, the blue boxplots show the permutation null distributions, the brown dots show the observed AUC score, and the blue and brown horizontal lines show the median (across all 30 distinct data splits) of the permutation null distributions and observed AUC scores, respectively.

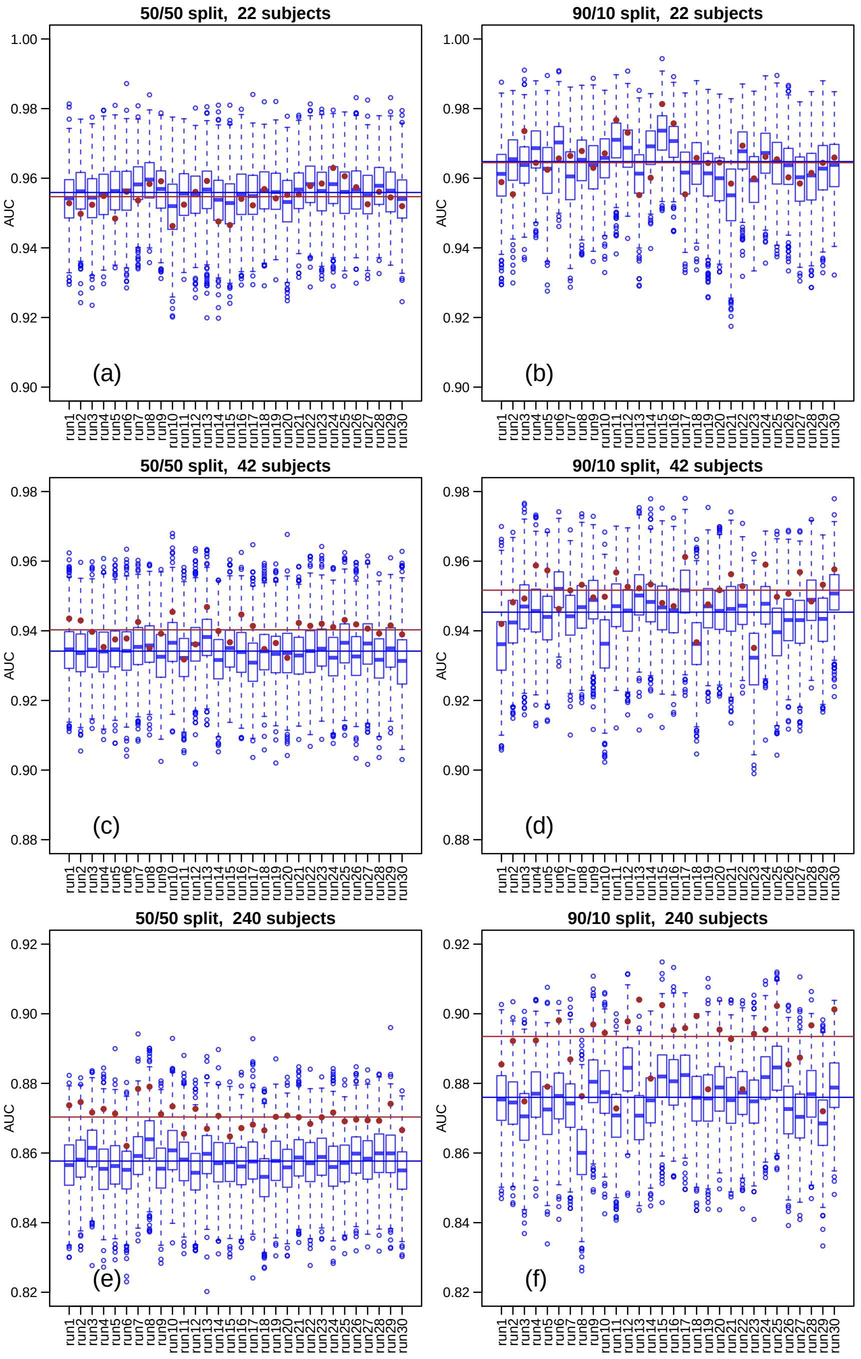

Figure 15: Comparison of record-wise data splits assigning 50% of the records to the training set and 50% to the text set (50/50 split) versus record-wise data splits assigning 90% of the records to the training set and 10% to the text set (90/10 split) for the mPower voice data across 30 distinct data splits. In all panels, the blue boxplots show the permutation null distributions, the brown dots show the observed AUC score, and the blue and brown horizontal lines show the median (across all 30 distinct data splits) of the permutation null distributions and observed AUC scores, respectively.
